# Anti-allergic Hydroxy Fatty Acids from *Typhonium blumei* Explored through ChemGPS-NP

**DOI:** 10.3389/fphar.2017.00356

**Published:** 2017-06-19

**Authors:** Michal Korinek, Yi-Hong Tsai, Mohamed El-Shazly, Kuei-Hung Lai, Anders Backlund, Shou-Fang Wu, Wan-Chun Lai, Tung-Ying Wu, Shu-Li Chen, Yang-Chang Wu, Yuan-Bin Cheng, Tsong-Long Hwang, Bing-Hung Chen, Fang-Rong Chang

**Affiliations:** ^1^Graduate Institute of Natural Products, College of Pharmacy, Kaohsiung Medical UniversityKaohsiung, Taiwan; ^2^Department of Biotechnology, College of Life Science, Kaohsiung Medical UniversityKaohsiung, Taiwan; ^3^Department of Pharmacognosy, Faculty of Pharmacy, Ain-Shams UniversityCairo, Egypt; ^4^Division of Pharmacognosy, Department of Medicinal Chemistry, Uppsala UniversityUppsala, Sweden; ^5^Natural Resource Development Institute of Pharmaceutics, Development Center for BiotechnologyNew Taipei City, Taiwan; ^6^Research Center for Natural Products and Drug Development, Kaohsiung Medical UniversityKaohsiung, Taiwan; ^7^Department of Medical Research, Kaohsiung Medical University HospitalKaohsiung, Taiwan; ^8^Center for Infectious Disease and Cancer Research, Kaohsiung Medical UniversityKaohsiung, Taiwan; ^9^Graduate Institute of Natural Products, College of Medicine, Chang Gung UniversityTaoyuan, Taiwan; ^10^Research Center for Chinese Herbal Medicine, Research Center for Food and Cosmetic Safety, and Graduate Institute of Health Industry Technology, College of Human Ecology, Chang Gung University of Science and TechnologyTaoyuan, Taiwan; ^11^Department of Anesthesiology, Chang Gung Memorial HospitalTaoyuan, Taiwan; ^12^The Institute of Biomedical Sciences, National Sun Yat-sen UniversityKaohsiung, Taiwan; ^13^Department of Marine Biotechnology and Resources, National Sun Yat-sen UniversityKaohsiung, Taiwan; ^14^Research Center for Environmental Medicine, Kaohsiung Medical UniversityKaohsiung, Taiwan; ^15^Cancer Center, Kaohsiung Medical University HospitalKaohsiung, Taiwan

**Keywords:** *Typhonium blumei*, hydroxy fatty acids, polyunsaturated fatty acids (PUFA), anti-allergic, anti-inflammatory, cytotoxic, ChemGPS-NP

## Abstract

Increasing prevalence of allergic diseases with an inadequate variety of treatment drives forward search for new alternative drugs. Fatty acids, abundant in nature, are regarded as important bioactive compounds and powerful nutrients playing an important role in lipid homeostasis and inflammation. Phytochemical study on *Typhonium blumei* Nicolson and Sivadasan (Araceae), a folk anti-cancer and anti-inflammatory medicine, yielded four oxygenated fatty acids, 12*R*-hydroxyoctadec-9*Z*,13*E*-dienoic acid methyl ester (**1**) and 10*R*-hydroxyoctadec-8*E*,12*Z*-dienoic acid methyl ester (**2**), 9*R*-hydroxy-10*E*-octadecenoic acid methyl ester (**3**), and 12R^*^-hydroxy-10*E*-octadecenoic acid methyl ester (**4**). Isolated compounds were identified by spectroscopic methods along with GC-MS analysis. Isolated fatty acids together with a series of saturated, unsaturated and oxygenated fatty acids were evaluated for their anti-inflammatory and anti-allergic activities *in vitro*. Unsaturated (including docosahexaenoic and eicosapentaenoic acids) as well as hydroxylated unsaturated fatty acids exerted strong anti-inflammatory activity in superoxide anion generation (IC_50_ 2.14–3.73 μM) and elastase release (IC_50_ 1.26–4.57 μM) assays. On the other hand, in the anti-allergic assays, the unsaturated fatty acids were inactive, while hydroxylated fatty acids showed promising inhibitory activity in A23187- and antigen-induced degranulation assays (e.g., 9*S*-hydroxy-10*E*,12*Z*-octadecadienoic acid, IC_50_ 92.4 and 49.7 μM, respectively). According to our results, the presence of a hydroxy group in the long chain did not influence the potent anti-inflammatory activity of free unsaturated acids. Nevertheless, hydroxylation of fatty acids (or their methyl esters) seems to be a key factor for the anti-allergic activity observed in the current study. Moreover, ChemGPS-NP was explored to predict the structure-activity relationship of fatty acids. The anti-allergic fatty acids formed different cluster distant from clinically used drugs. The bioactivity of *T. blumei*, which is historically utilized in folk medicine, might be related to the content of fatty acids and their metabolites.

## Introduction

Inflammation is a crucial part of an organism immunologic response to defend itself from invading pathogens and maintain homeostasis. However, pathological inflammatory responses or allergic reactions can cause a substantial burden to human health. For instance, prolonged inflammation plays an important role in various diseases including allergy, cardiovascular diseases and carcinogenesis (Shacter and Weitzman, 2002; Grivennikov et al., 2010). Neutrophils play an essential role in the non-specific (innate) immune system against invading pathogens and are usually the first cells recruited to inflammatory sites. Uncontrolled activation of neutrophils will trigger pathological degranulation and respiratory burst, leading to tissue damage, a hallmark associated with many inflammatory diseases (Korkmaz et al., 2010). Therefore, elastase levels, as well as the production of superoxide, serve as important markers of inflammation in human neutrophils (Tsai et al., 2015).

Allergic disorders, such as allergic rhinitis, allergic conjunctivitis, asthma, atopic eczema, or food allergies, are caused by pathological hypersensitivity reactions initiated by immunologic mechanisms (Johansson et al., 2001). Mast cells play a crucial role in triggering allergic response via IgE-mediated degranulation. The *in vitro* model, detecting the release of β-hexosaminidase or histamine by RBL-2H3 cells, has been regarded as an useful indicator to evaluate the activation of mast cells in various acute allergic and inflammatory responses (Chen et al., 2009). Over the last century, several classes of anti-allergic and anti-inflammatory agents were developed. Natural compounds proved effective as drug leads fighting inflammatory reactions, however, they did not show such effect against allergic conditions (Newman and Cragg, 2016). The untapped potential of natural products as an inexhaustible mine of biologically active compounds along with their excellent safety profile encouraged us to investigate the potential of separating natural products with promising anti-allergic activity.

*Typhonium* is a genus of tropical plants, which belongs to family Araceae and comprises about 40 species. Several *Typhonium* species are often confused, four of them, namely *Typhonium blumei* (= *Typhonium divaricatum*), *Typhonium roxburghii, Typhonium flagelliforme*, and *Typhonium trilobatum*, were critically revised by Nicolson and Sivadasan (1981). *Typhonium* species are widely used as traditional and folk medicines to treat a myriad of ailments. For example, *T. giganteum* is a traditional Chinese medicine (TCM) used as a part of various formulas to treat respiratory diseases, stroke, epilepsy and nowadays applied in cancer treatment (Chang, 1992). Another species common in Malaysia, *T. flagelliforme*, has been extensively studied for its anticancer activity based on its folk medicinal use (Lai et al., 2008). Additionally, *T. flagelliforme* extracts were reported to exhibit anti-asthmatic, anti-inflammatory and analgesic effects (Zhong et al., 2001).

*Typhonium blumei* Nicolson and Sivadasan (= *T. divaricatum*, in Chinese Tu-Ban-Xia), is a wildly growing weed native to South and Southeast Asia, and naturalized in Australia and Africa (Wang and Yang, 1996; Huang, 2000). In Taiwan, *T. blumei* is consumed in order to treat cancer such as leukemia or liver cancer, cough, swelling, and snake bites, and it is used topically to treat bruises and skin cancer (Li, 2006). Phytochemical and biological studies on *T. blumei* are scanty. Antiviral mannose-binding lectin has been isolated from this plant (Kong et al., 2006; Luo et al., 2007). A cytotoxic study on *T. blumei* crude extract revealed the presence of lipids and steroids using GC-MS analysis (Hsu et al., 2011). Recently, we showed that the nonpolar extracts of *T. blumei* and *T. roxburghii* exhibited potent anti-allergic activity through affecting the calcium signaling pathway (Korinek et al., 2016a). Our results suggested that the saturated and unsaturated fatty acid content of the nonpolar extracts is responsible for such activity. However, the specific components of *T. blumei* nonpolar extracts responsible for the anti-allergic activity have never been revealed.

Fatty acids, particularly unsaturated essential fatty acids with proper ratio of n-6 to n-3, represent interesting bioactive compounds and powerful nutrients that play an important role in lipid homeostasis and in the prevention of cardiovascular diseases (Rajaram, 2014; Maehre et al., 2015; Vangaveti et al., 2016; Xu et al., 2016). An imbalance caused by excessive intake of n-6 fatty acids from the modern Western diet may contribute to several chronic diseases (Lopez-Vicario et al., 2016). Therefore, higher intake of n-3 fatty acids is recommended (Calder, 2008; Beermann et al., 2016; Michalak et al., 2016). The biological properties of n-3 fatty acids have been extensively studied and their beneficial effects may be due, in part, to their suppressive activity on inflammatory processes (Hwang et al., 2009; Calder, 2015). Recently, it was found that the metabolism of n-3 fatty acids (such as docosahexaenoic, DHA, and eicosapentaenoic, EPA) gives rise to oxygenated mediators (i.e., oxygenated fatty acid derivatives), called resolvins, protectins, and maresins, which possess considerable inflammation resolving properties (Lundstrom et al., 2013). Several mechanisms underlying the anti-inflammatory effect of n-3 fatty acids and their oxidative metabolites were reported. Those mechanisms include the alteration of the fatty acid composition of cell membrane phospholipids, disruption of lipid rafts and modulation of T-cell function. Modulation of the expression of some inflammation-related genes is also involved through the alteration of transcription factors activity, such as nuclear factor kappa B (NF-κB), and peroxisome proliferator-activated receptor (PPARγ) (Calder, 2015).

The growing evidence on allergy preventive (Willemsen, 2016), anti-inflammatory (Calder, 2015; Michalak et al., 2016), and cytotoxic (Jing et al., 2013) effects of fatty acids, led us to conduct a study aiming to understand the structural requirements of these activities, using *in vitro* cell line models. We performed a biological and phytochemical study on *T. blumei* and discussed the isolation of four hydroxylated fatty acids. We also evaluated the cytotoxic, anti-inflammatory and anti-allergic activities of a series of saturated, unsaturated and hydroxylated fatty acids.

## Materials and methods

### Chemicals and reagents

Optical rotations were achieved by a JASCO P-2000 digital polarimeter (JASCO Inc., Tokyo, Japan). JASCO V-570 UV/vis/NIR spectrophotometer (JASCO Inc., Tokyo, Japan) was used to measure UV spectra. CD spectra were measured on a JASCO J-810 spectropolarimeter (JASCO Inc., Tokyo, Japan). IR spectra were obtained on an FT/IR-4600 JASCO spectrophotometer (JASCO Inc., Tokyo, Japan). NMR spectra were obtained by Varian-Mercury-plus 400 MHz FT-NMR (Varian Inc., Palo Alto, CA, USA) and JEOL JNM-ECS 400 MHz NMR spectrometer (JEOL Ltd., Tokyo, Japan). Sephadex LH-20 (Merck KGaA, Darmstadt, Germany) and silica gel (Kieselgel 60, 70–230 and 230–400 mesh, Merck KGaA, Darmstadt, Germany) were used for column chromatography. TLC analyses were carried out on silica gel pre-coated (Kieselgel 60 F_254_ and RP-18 F_254s_, Merck KGaA, Darmstadt, Germany). Spots on TLC plates were detected using 50%_(aq)_H_2_SO_4_ followed by heating on a hot plate. Gas chromatography-mass spectrometry analyses were performed on DSQ II Single Quadrupole GC/MS (Thermo Fisher Scientific Inc., Waltham, MA, USA) using DB-5MS and HP-5MS capillary columns (30 m × 0.25 mm, with 0.25 μm coating, Agilent, J & W Scientific, Santa Clara, CA, USA). The RP-HPLC analysis was performed using Shimadzu LC-10AT instrument equipped with Shimadzu RIP-10A refractive index detector (Shimadzu Inc., Kyoto, Japan) using C-18 column (20 × 250 mm, Cosmosil). Dulbecco's modified Eagle's medium (high glucose) powder (DMEM), 3-(4,5-dimethylthiazol-2-yl)-2,5-diphenyltetrazolium bromide (MTT), *p*-nitrophenyl-*N*-acetyl-d-glucosaminide (*p*-NAG), penicillin and streptomycin, genistein, dexamethasone, doxorubicin hydrochloride, calcium ionophore A23187, and dimethyl sulfoxide (DMSO) were purchased from Sigma-Aldrich (St. Louis, MO, USA). Fetal bovine serum (FBS) was obtained from Hyclone (Logan, UT, USA). Mouse anti-DNP IgE (mIgE-DNP) antibody was a generous gift from Dr. Daniel H. Conrad (Virginia Commonwealth University, Richmond, VA, USA). Dinitrophenyl-conjugated bovine serum albumin (DNP-BSA) was purchased from Pierce (Rockford, IL, USA). Stearic acid, stearic acid methyl ester, palmitic acid, palmitic acid methyl ester, oleic acid, oleic acid methyl ester, *cis*-vaccenic acid methyl ester, petroselinic acid methyl ester, and γ-linolenic acid were obtained from TCI (Tokyo Chemical Industry Co., Ltd., Japan); undecylenic acid, undecylenic acid methyl ester, *cis*-vaccenic acid, petroselinic acid, conjugated (9*Z*,11*E*)-linoleic acid, *cis*-5,8,11,14,17-eicosapentaenoic acid (EPA), *cis*-4,7,10,13,16,19-docosahexaenoic acid (DHA), 2-hydroxystearic acid, 12-hydroxystearic acid methyl ester, 12-oxostearic acid methyl ester, 2-hydroxy-9*Z*-octadecenoic acid (2OHOA), ricinoleic acid, ricinoleic acid methyl ester, ricinelaidic acid, 9*S*-hydroxy-10*E*,12*Z*-octadecadienoic acid (9(S)-HODE) from Sigma-Aldrich; linoleic acid, α-linolenic acid, α-linolenic acid methyl ester from Acros Organics (Thermo Fisher Scientific Inc., Geel, Belgium); linoleic acid methyl ester, 12-hydroxystearic acid from Alfa Aesar (Thermo Fisher Scientific Inc., Heysham, Lancashire, United Kingdom). All other chemicals and reagents were purchased at the highest purity and quality possible.

### Plant materials

*Typhonium blumei* was collected from Nantou county, Taiwan, in September 2009. Botanical identification of the plant was made by Professor Ming-Hong Yen of Kaohsiung Medical University, Taiwan. Voucher specimen no. KMU-TB1 was deposited at Kaohsiung Medical University, Taiwan.

### Extraction and isolation

*Typhonium blumei* plant material was washed by tap water and separated into leaves (aerial) and rhizomes (underground part). Leaves were cut and extracted with methanol (2 l × 3, 24 h). Extracts were filtrated and evaporated to dryness under reduced pressure using rotary evaporator and vacuum drying oven. The methanol extract of the leaves was partitioned between dichloromethane and water. *Typhonium blumei* rhizomes (423 g) were chipped, air-dried, and extracted repeatedly with methanol (2 l × 3, 24 h) at room temperature. The combined methanol extracts were then evaporated. The methanol extract (14.4 g) was partitioned between ethyl acetate and water. The sample of non-polar fractions, dichloromethane for leaves (100 mg) and ethyl acetate for rhizomes (100 mg) were further partitioned using n-hexane and methanol. The corresponding partition fractions were collected, filtered and evaporated to dryness. Filtered ethyl acetate layer (1.32 g) was subjected to a silica gel column chromatography (95 g, 70–230 mesh) eluted with *n*-hexane/dichloromethane (4:1) gradually changed to dichloromethane/methanol (5:1) and separated into 13 fractions according to TLC analysis. A mixture C (stigmasterol and β-sitosterol, 9 mg) was precipitated from fraction 5 using *n*-hexane. Fr. 5 (47 mg) was subjected to silica gel column chromatography eluted with solvent system *n*-hexane/ethyl acetate (10:1) gradually changed to ethyl acetate/methanol (4:1) furnishing 13 fractions. Fr. 5-6 was identified as a mixture A (palmitic acid methyl ester, stearic acid methyl ester, oleic acid methyl ester, margaric acid methyl ester, pentadecanoic acid methyl ester, 8 mg, GC-MS). Fr. 5-10 was purified using preparative TLC eluted with dichloromethane/*n*-hexane (100:1) and yielded a mixture B (β-sitost-4-en-3-one, stigmasta-4,22-dien-3-one, campest-4-en-3-one, 2.5 mg, GC-MS). Fraction 7 (128 mg) was eluted with dichloromethane/methanol (1:1) on Sephadex LH-20 column. Fraction 7-2 was subjected to NP-SPE eluted with *n*-hexane/ethyl acetate (15:1 yielding fr.1, 10:1 fr.2, 7:1 fr.3, 3:1 fr.4) and washed with methanol (fr.5). Fractions 7-2-2 and 7-2-3 were subsequently subjected to RP-HPLC chromatography (Shimadzu RID-10A, 20 × 250, 5 ml/min) eluted by acetonitrile/water (72:28) yielding compound **1** (12*R*-hydroxy-9*Z*,13*E*-octadecadienoic acid methyl ester, 2.4 mg), compound **2** (10*R*-hydroxy-8*E*,12*Z*-octadecadienoic acid methyl ester, 3.5 mg), compound **3** (9*R*-hydroxy-10*E*-octadecenoic acid methyl ester, 1 mg) and compound **4** (12R^*^-hydroxy-10*E*-octadecenoic acid methyl ester, 0.6 mg). Precipitation of fraction 9 yielded a mixture D (stigmasterol-3-*O*-β-D-glucoside, β-sitosterol-3-*O*-β-D-glucoside, 1 mg). The isolated compounds were identified by spectroscopic data and GC-MS analyses, comparing the mass spectra with data from *Wiley/NBS Registry of Mass Spectral Data* (version 5.0)/ *National Institute of Standards and Technology (NIST) Library MS Search* (version 2.0) or literature.

### Experimental

#### 12*R*-Hydroxy-9*Z*,13*E*-octadecadienoic acid methyl ester (1, 2.4 mg)

${\left[\alpha \right]}_{\text{D}}^{25}-$257 (*c* 0.06, EtOH); UV (EtOH) λ_max_ (log ε) 226 (3.15), 236 (3.15), 275 (2.77) nm; CD (c 5 mM, EtOH) λ_max_ (Δε_max_) 203 (0.05), 246.5 (−0.07) nm; IR (neat) ν_max_ 3428 (OH), 2925, 2855, 1737 (C = O), 1462 (C = C), 1285, 1038, 805 cm^−1^; ^1^H NMR (CD_3_OD, 400 MHz) and ^13^C NMR (CD_3_OD, 100 MHz): see Table 1, GC-EIMS (Figure S7) *m*/*z* (rel. intensity); 292 [M^+^ − H_2_O] (2), 198 (2), 166 (10), 124 (11), 113 (73), 95 (59), 81 (17),69 (24), 67 (26), 57 (100). Mosher's esterification reaction (MTPA chloride, DCM, crystal of DMAP, 40°C, overnight) led to decomposition yielding decarboxylated product.

**Table 1:** ^1^H NMR (400 MHz) and ^13^C NMR (100 MHz) data of isolated compounds.

**Position**	**1^a^ δ_H_ ppm (mult., *J* in Hz)**	**2^a^ δ_H_ ppm (mult., *J* in Hz)**	**3^b^ δ_H_ ppm (mult., *J* in Hz)**	**4^b^ δ_H_ ppm (mult., *J* in Hz)**	**1^a^ δ^C^ ppm**	**2^a^ δ^C^ ppm**
1					176.0 C	176.0 C
2	2.32, t (7.2)	2.31, t (7.2)	2.30, t (7.6)	2.30, t (7.6)	34.8 CH_2_	34.8 CH_2_
3	1.60, m	1.60, m	1.60, m	1.58, m	26.0 CH_2_	26.0 CH_2_
4	1.32, m	1.32, m	1.32, m	1.32, m	30.2 CH_2_	29.8 CH_2_
5	1.32, m	1.32, m	1.32, m	1.32, m	30.2 CH_2_	30.0 CH_2_
6	1.32, m	1.32, m	1.32, m	1.32, m	30.3 CH_2_	32.7 CH_2_
7	1.32, m	2.04, m	1.32, m	1.32, m	30.6 CH_2_	33.2 CH_2_
8	2.04, q (6.4)	5.62, dt (15.2, 6.8)	1.47, m	1.32, m	28.4 CH_2_	132.7 CH
9	5.44, m	5.44, m	4.03, q (6.4)	2.02, q (6.8)	132.6 CH	134.0 CH
10	5.44, m	3.99, q (6.8)	5.44, dd (15.2, 6.8)	5.62, dtd (15.2, 6.8, 3.2)	126.4 CH	73.7 CH
11a	2.27, t (6.4)	2.27, t (6.4)	5.62, dtd (15.2, 6.8, 4.4)	5.44, dd (15.2, 7.2)	36.5 CH_2_	36.4 CH_2_
11b	2.20, m	2.20, m				
12	3.99, q (6.8)	5.44, m	2.02, q (6.8)	4.03, q (6.4)	73.7 CH	126.4 CH
13	5.44, m	5.44, m	1.32, m	1.47, m^c^	133.9 CH	132.4 CH
14	5.62, dt (15.2, 6.4)	2.05, m	1.32, m	1.32, m	132.6 CH	28.4 CH_2_
15	2.05, q (6.4)	1.32, m	1.32, m	1.32, m	33.0 CH_2_	30.4 CH_2_
16	1.32, m	1.32, m	1.32, m	1.32, m	32.6 CH_2_	30.2 CH_2_
17	1.32, m	1.32, m	1.32, m	1.32, m	23.2 CH_2_	23.6 CH_2_
18	0.91, t (7.2)	0.91, t (6.8)	0.88, t (6.4)	0.88, t (6.4)	14.3 CH_2_	14.4 CH_2_
−OCH_3_	3.65, s	3.65, s	3.66, s	3.66, s	52.0 CH_3_	52.0 CH_3_

a *Compounds **1** and **2** were dissolved in CD_3_OD*.

b *Compounds **3** and **4** in CDCl_3_*.

c *The signal is overlapping with H_2_O*.

#### 10*R*-Hydroxy-8*E*,12*Z*-octadecadienoic acid methyl ester (2, 3.5 mg)

${\left[\alpha \right]}_{\text{D}}^{25}$−266 (*c* 0.06, EtOH); UV (EtOH) λ_max_ (log ε) 226 (3.17), 278 (2.92) nm; CD (c 5 mM, EtOH) λ_max_ (Δε_max_) 190.5 (+0.01), 236 (−0.08) nm; IR (neat) ν_max_ 3415 (OH), 2925, 2855, 1734 (C = O), 1437 (C = C), 1262, 1014, 736 cm^−1^; ^1^H NMR (CD_3_OD, 400 MHz) and ^13^C NMR (CD_3_OD, 100 MHz): see Table 1; GC-EIMS (Figure S14) *m*/*z* (rel. intensity); 292 [M^+^ − H_2_O] (1), 199 (27), 181 (6), 167 (100), 149 (28), 139 (40), 121 (75), 83 (37), 69 (20), 67 (16), 57 (32).

#### 9*R*-Hydroxy-10*E*-octadecenoic acid methyl ester (3, 1.0 mg)

${\left[\alpha \right]}_{\text{D}}^{25}$−386 (*c* 0.01, EtOH); UV (EtOH) λ_max_ (log ε) 244 (2.84), 273 (2.75) nm; IR (neat) ν_max_ 3452 (OH), 2925, 2855, 1742 (C = O), 1460 (C = C), 1261, 1036, 806 cm^−1^; ^1^H NMR (CDCl_3_, 400 MHz): see Table 1; GC-EIMS (Figure S15) *m*/*z* (rel. intensity); 294 [M^+^ − H_2_O] (10), 213 (4), 181 (8), 155 (34), 121 (20), 95 (48), 81 (82), 67 (84), 55 (93), 43 (100).

#### 12R^*^-Hydroxy-10*E*-octadecenoic acid methyl ester (4, 0.6 mg)

${\left[\alpha \right]}_{\text{D}}^{25}$−80 (*c* 0.015, EtOH); UV (EtOH) λ_*max*_ (log ε) 244 (2.84), 273 (2.75) nm; IR (neat) ν_max_ 3404 (OH), 2922, 2852, 1680 (C = O), 1463 (C = C), 1260, 1028, 798 cm^−1^; ^1^H NMR (CDCl_3_, 400 MHz): see Table 1; GC-EIMS (Figure S16) *m*/*z* (rel. intensity); 294 [M^+^ − H_2_O] (2), 227 (10), 195 (36), 149 (20), 127 (34), 109 (46), 81 (68), 67 (80), 55 (100).

#### Mixture A (9 mg)

GC-MS analysis (palmitic acid methyl ester 64.2%, stearic acid methyl ester 18.0%, oleic acid methyl ester 12.3%, margaric acid methyl ester 3.6%, pentadecanoic acid methyl ester 1.9%; see Supporting Information, Figure S17).

#### Mixture B (2.5 mg)

GC-MS analysis (β-sitost-4-en-3-one 51.2%, stigmasta-4,22-dien-3-one 26.0%, campest-4-en-3-one 22.8%; see Supporting Information, Figure S18).

### Cell culture

The mucosal mast cell-derived rat basophilic leukemia (RBL-2H3) cell line was purchased from Bioresource Collection and Research Center (Hsin-Chu, Taiwan). Cells were grown in DMEM supplemented with 10% FBS and 100 U/ml penicillin plus 100 μg/ml streptomycin. Cells were cultured in 10 cm cell culture dishes (Cellstar) at 37°C in a humidified chamber with 5% CO_2_ in the air. Cells were subcultured using trypsin at 80% confluency and were plated at 2 × 10^5^ cells/ml in culture plates for the secretion assays.

### Cell viability assay

A methyl thiazolyl tetrazolium (MTT) assay was used to measure potential toxic effects of samples on RBL-2H3 cells (Korinek et al., 2016b). The method is based on the cleavage of the tetrazolium rings of pale yellow MTT by mitochondrial dehydrogenase enzyme from viable cells forming crystals of dark blue formazan accumulated in healthy cells. Briefly, RBL-2H3 cells at a concentration of 2 × 10^4^ cells/well were seeded in 96 wells plate overnight. Cells were washed with PBS (phosphate buffer saline) and treated with various concentrations of samples (dissolved in DMSO) or untreated control (0.5% DMSO in medium). After 24 h of incubation at 37°C in 5% CO_2_, the medium was removed from each well. A stock MTT solution (5 mg/ml) was diluted 1:10 in culture medium and was added to wells (100 μl per well). Cells were incubated at 37°C in 5% CO_2_ for 1 h. The medium was removed and formed formazan crystals were dissolved in DMSO (100 μl). The plate was gently shaken and the absorbance at 550 nm was measured using microplate reader. The degree of cell viability of each sample was calculated as the percentage of control value (untreated cells). Maximal tolerated dose of DMSO was 0.5%. It served as a positive control not affecting RBL-2H3 cells growth. Triton X-100 (0.5% solution) was used as positive control causing the death of all cells in a well.

### Degranulation β-hexosaminidase assay induced by A23187 or antigen

The degree of A23187-induced and antigen-induced degranulation in RBL-2H3 cells was determined by a β-hexosaminidase activity assay as previously described (Korinek et al., 2016b) with some modifications. Briefly, RBL-2H3 cells were dispensed into the 96-wells plate at a density of 2 × 10^4^ cells/well. Cells were incubated at 37°C in 5% CO_2_ for at least 5 h to allow cells complete adherence to the bottom of the wells. Cells were washed with PBS and various concentrations of samples (dissolved in DMSO) or medium (0.5% DMSO, untreated control) were added to each well (100 μl), followed by 20 h of incubation at 37°C in 5% CO_2_. Then the cells were washed twice by pre-warmed Tyrode's buffer (135 mM NaCl, 5 mM KCl, 1.8 mM CaCl_2_, 1.0 mM MgCl_2_, 5.6 mM glucose, 20 mM HEPES, and 1 mg/ml BSA at pH 7.4). The cells for the antigen-induced experiment were first sensitized with anti-DNP IgE (2 μg/ml) for at least 2 h, followed by thorough washing by pre-warmed Tyrode's buffer. The cells were stimulated by calcium ionophore A23187 (1 μM, A23187-induced) or with the cross-linking antigen DNP-BSA (100 ng/ml, antigen-induced). The cells were incubated at 37°C in 5% CO_2_ for 1 h. Unstimulated cells were either lysed with 0.5% Triton X-100 solution for the total amount of β-hexosaminidase release or left untreated for spontaneous release of β-hexosaminidase. Stimulated but untreated cells served as control. Then aliquots of supernatants (50 μl) collected from the control and experimental wells were incubated with an equal volume (50 μl) of 1 μM of *p*-NAG prepared in 0.1 M citrate buffer (pH 4.5) serving as a substrate for released β-hexosaminidase. After 1 h of incubation at 37°C, the reaction was quenched by the addition of 100 μl of stop buffer (0.1 M Na_2_/NaHCO_3_, pH 10.0). Absorbance was measured at 405 nm on a microplate reader. The inhibition percentage of β-hexosaminidase release from RBL-2H3 cells was calculated as the percentage of control value (untreated stimulated cells). Dexamethasone was used as a positive control.

### Measurement of platelet aggregation

Human blood was obtained from healthy human volunteers and the coagulation was prevented by addition of acid citrate dextrose. The platelet suspension was washed and further processed as previously described (Wei et al., 2015). Platelets were then suspended in Tyrode's solution (containing 2 mM Ca^2+^, 11.1 mM glucose and 3.5 mg/ml bovine serum albumin) at a concentration of 3 × 10^8^ platelets/ml. Platelet aggregation was measured turbidimetrically using light-transmission aggregometer (Chrono-Log, Havertown, PA, USA; Wu et al., 2004). Briefly, the platelet suspension was incubated with samples or with dimethyl sulfoxide (DMSO, vehicle) for 3 min while stirring at 1,200 rpm, followed by the addition of platelet aggregation inducers (collagen at 10 μg/ml or thrombin 0.05 U/ml). The level of platelet aggregation was represented by the maximal increase of light transmission within 5 min after the addition of inducer.

### Preparation of human neutrophils

Human neutrophils were obtained from venous blood of healthy, adult volunteers (20–30 years old) using a protocol approved by the Institutional Review Board at Chang Gung Memorial Hospital (protocol number 102-1595A3). Human neutrophils were isolated using a standard method of dextran sedimentation prior to centrifugation in a Ficoll-Hypaque gradient and hypotonic lysis of erythrocytes (Boyum, 1968). Purified neutrophils containing >98% viable cells, as determined by the trypan-blue exclusion method (Jauregui et al., 1981), were resuspended in a Ca^2+^-free Hank's buffered salt solution (HBSS) at pH 7.4 and were maintained at 4°C prior to use.

### Superoxide anion generation assay

Neutrophil superoxide anion generation was based on the superoxide dismutase (SOD)-inhibitable reduction of ferricytochrome *c* according to described procedures (Hwang et al., 2006; Yang et al., 2013). Briefly, after supplementation with 0.5 mg/ml ferricytochrome *c* and 1 mM Ca^2+^, neutrophils (6 × 10^5^/ml) were equilibrated at 37°C for 2 min and incubated with different concentrations of fractions or DMSO (as control) for 5 min. Cells were incubated with cytochalasin B (CB; 1 μg/ml) for 3 min prior to the activation with formyl-methionyl-leucyl-phenylalanine (fMLF; 100 nM) for 10 min. Changes in absorbance with the reduction of ferricytochrome *c* at 550 nm were continuously monitored in a double-beam, six-cell positioner spectrophotometer with constant stirring (Hitachi U-3010, Tokyo, Japan). Calculations were based on the differences in the reactions with and without SOD (100 U/ml) divided by the extinction coefficient for the reduction of ferricytochrome *c* (21.1 mM^−1^ cm^−1^). Genistein was used as the positive control.

### Elastase release inhibition assay

Degranulation of azurophilic granules was determined by measuring the elastase release as described previously (Yang et al., 2013). Experiments were performed using MeOSuc-Ala-Ala-Pro-Val-*p*-nitroanilide as the elastase substrate. After supplementation with MeOSuc-Ala-Ala-Pro-Val-*p*-nitroanilide (100 μM), neutrophils (6 × 10^5^/ml) were equilibrated at 37°C for 2 min and incubated with fractions for 5 min. Cells were stimulated with fMLF (100 nM)/CB (0.5 μg/ml), and changes in the absorbance at 405 nm were monitored continuously in order to measure the elastase release. The results were expressed as the percent of elastase release in the fMLF/CB-activated, drug-free control system. Genistein was used as the positive control.

### Cytotoxicity assay

MTT viability assay was used according to a previously described method (Lai et al., 2013). HepG2 (1 × 10^4^ cells), A549 (5 × 10^3^ cells), and MDA-MB-231 (1 × 10^4^ cells) were seeded into 96-well plates. The cells were treated with the test compounds for 72 h. The medium was removed from wells and 100 μl of MTT solution (0.5 mg/ml) were added. The plates were incubated at 37°C for 1 h to form formazan crystals. The crystals were dissolved in DMSO (100 μl) and plates were gently shaken. Absorbance at 550 nm was measured using microplate reader. The degree of cell viability of each sample was calculated as the percentage of control value (untreated cells). Doxorubicin hydrochloride was used as a positive control.

### GC-MS analysis

The samples were filtered through 0.22 μm filter and subjected to GC-MS (gas chromatography-mass spectrometry) analysis using DSQ II Single Quadrupole GC/MS (Thermo Fisher Scientific, USA). The samples were vaporized at 250°C in standard split mode (1:50) and separated on DB-5MS and HP-5MS capillary columns (30 m × 0.25 mm, with 0.25 μm coating, Agilent, J & W Scientific, Santa Clara, CA, USA). The column oven temperature program for compound 1 was set as follows, initial 200°C, held for 10 min, increased to 300°C at 10°C/min, held for 5 min. For compound 2, initial 100°C was held for 5 min, increased to 200°C at 50°C/min, held for 5 min, then increased to 300°C at 50°C/min and held for 5 min. For compounds **3** and **4**, the column oven temperature was maintained at 70°C, held for 3 min, then increased to 180°C at 20°C/min, held for 5 min, then increased to 280°C at 5°C/min and held for 5 min. For mixtures A and B, the initial temperature was 100°C, held for 10 min, then increased to 300°C by 20°C/min and held for 10 min. Helium gas carrier flow was set to 1 ml/min. The interface and ion source temperature were adjusted to 250°C. Electron impact ionization of 70 eV was utilized. Injection volume was 1 μl and the mass range was *m*/*z* 45–800. Identification of the compounds was based on a comparison of the mass spectra with data from *Wiley/NBS Registry of Mass Spectral Data* (version 5.0)/*National Institute of Standards and Technology (NIST) MS Search* (version 2.0). The relative percentage of each compound in mixtures A and B was quantified based on the peak area integrated by the analysis program.

### ChemGPS-NP analysis

ChemGPS-NP (http://chemgps.bmc.uu.se) is principal component analysis (PCA)-based model that serves as a tool for navigation in biologically relevant chemical space. It is composed of eight principal components (PCs) based on 35 chemical descriptors, which represent physico-chemical properties such as size, shape, flexibility, rigidity, polarizability, lipophilicity, polarity, and hydrogen bond capacity. PC1 represents size, shape, and polarizability (red axis), PC2 aromatic- and conjugation-related properties (orange axis), and PC3 lipophilicity, polarity, and H-bond capacity (green axis). ChemGPS-NP_Web_ (Rosen et al., 2009) (http://chemgps.bmc.uu.se) online tool was used to calculate the prediction scores based on the structural information of fatty acids derived from SMILES using the ChemBioDraw software. The following clinically used anti-allergic drugs were plotted into chemical space: glucocorticoids (black squares; dexamethasone, triamcinolone, budesonide, fluticasone), immunosuppressants (white squares; pimecrolimus, tacrolimus), leukotriene inhibitors (gray squares; pranlukast, zafirlukast, montelukast), mast cell stabilizers (orange squares; lodoxamide, nedocromil, cromolyn), antihistaminics (brown squares; chlorpheniramine, diphenhydramine, dimetindene, clemastine, loratadine, cetirizine, bilastine, fexofenadine) and “dual antihistaminics” known to inhibit mast cell degranulation (red squares; ketotifen, azelastine, rupatadine). The drugs are listed by increasing value of PC1 (red axis) within each group. All compounds were then plotted into ChemGPS-NP chemical property space using the software Grapher 2.5 (Mac OS).

### Statistics

The results were expressed as mean ± SD value of three independent determinations unless otherwise specified. The IC_50_ values were calculated using the Microsoft Office (linear function). Statistical significance was calculated by one-way analysis of variance (ANOVA), followed by Dunnett's test (SigmaPlot, Systat Software Inc., San Jose, CA, USA). Values with ^*^p < 0.05, ^**^p < 0.001 were considered statistically significant.

## Results

### Bioactivity screening of *Typhonium blumei* (TB)

**Figure 1:**
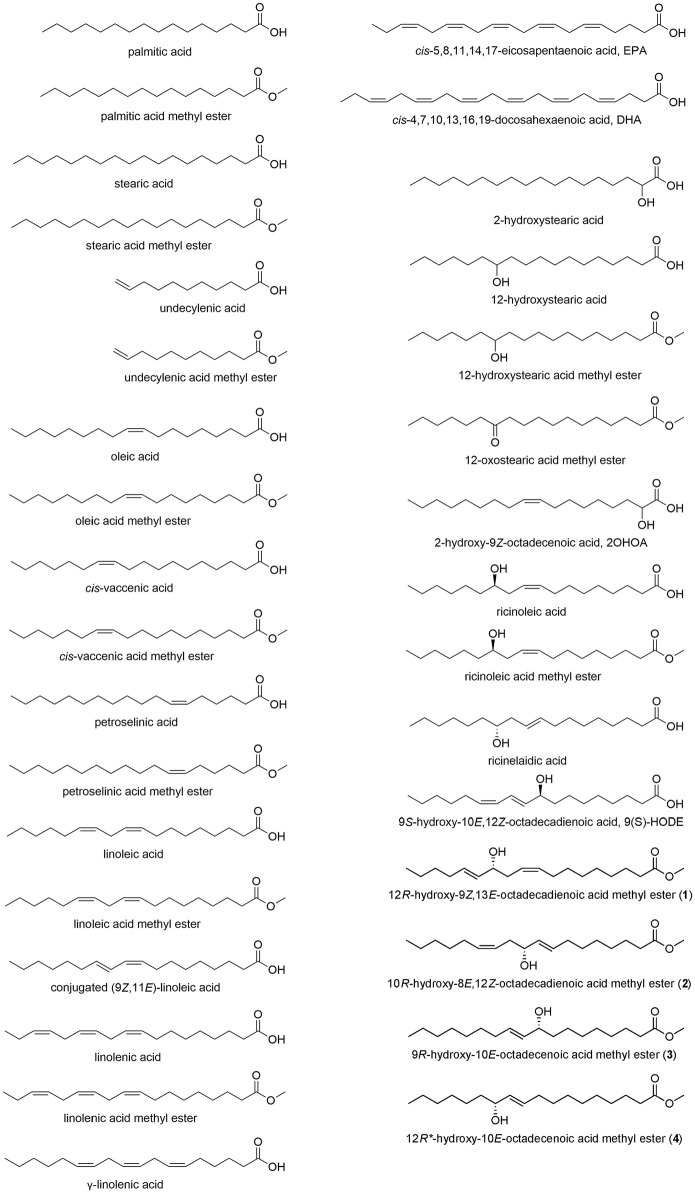
Structures of fatty acids, including the hydroxy fatty acids 1–4 isolated from *Typhonium blumei*.

TB plant was separated into leaves (aerial) and rhizomes (underground) and extracted with methanol. The crude extracts and partition fractions were screened for anti-platelet, cytotoxic, anti-inflammatory, and anti-allergic activities. The results showed that the polar butanol layer of TB leaves exhibited 100% inhibitory activity on aggregation induced by either collagen or thrombin at 50 μg/ml (Table S2). On the other hand, the nonpolar dichloromethane (TB leaves) or ethyl acetate (TB rhizomes) layers inhibited specifically collagen-induced aggregation by 93.6 and 42.9%, respectively (Table S2). Cytotoxicity screening on six cancer cell lines showed the cytotoxic effect of the nonpolar dichloromethane (TB leaves) and ethyl acetate (TB rhizomes) layers against hepatocellular carcinoma cells, HepG2 (49.6 and 51.7% at 20 μg/ml, respectively, Table S3). Partition fractions, except for the polar water layers, exhibited significant anti-inflammatory activity by inhibiting superoxide anion generation and elastase release in human neutrophils reaching up to 100% inhibition at 10 μg/ml (Table S4). The ethyl acetate layer (TB rhizomes) was shown to exert anti-allergic activities inhibiting A23187-induced β-hexosaminidase release with IC_50_ 94.5 μg/ml (Korinek et al., 2016a). The active nonpolar ethyl acetate layer of TB rhizomes was further separated using silica gel column chromatography to yield 13 fractions, TB-1 to TB-13. Among these fractions, fractions TB-7 and TB-9 demonstrated cytotoxic activity toward HepG2 cells with 33.5 and 56.8% inhibition, respectively (Table S5). Certain fractions showed anti-inflammatory activity including TB-2, TB-8 to TB-11, and TB-13 that inhibited superoxide anion generation (IC_50_ values 1.45–6.5 μg/ml) and TB-1, TB-2 and TB-7 to TB-13 that inhibited elastase release (IC_50_ 1.31–6.42 μg/ml) in human neutrophils (Table S6). Also, several fractions showed anti-allergic activity including TB-2, TB-4, TB-7, and TB-11 that inhibited A23187-induced degranulation of mast cells (25.7–41.0% at 100 μg/ml), while TB-9 and TB-10 showed 42.7 and 42.0% inhibition of degranulation at nontoxic concentration of 50 μg/ml in RBL-2H3 cells (Table S7). The purification of TB-7 led to the isolation of four hydroxylated fatty acids (Figure 1).

### Structure elucidation

**Figure 2:**
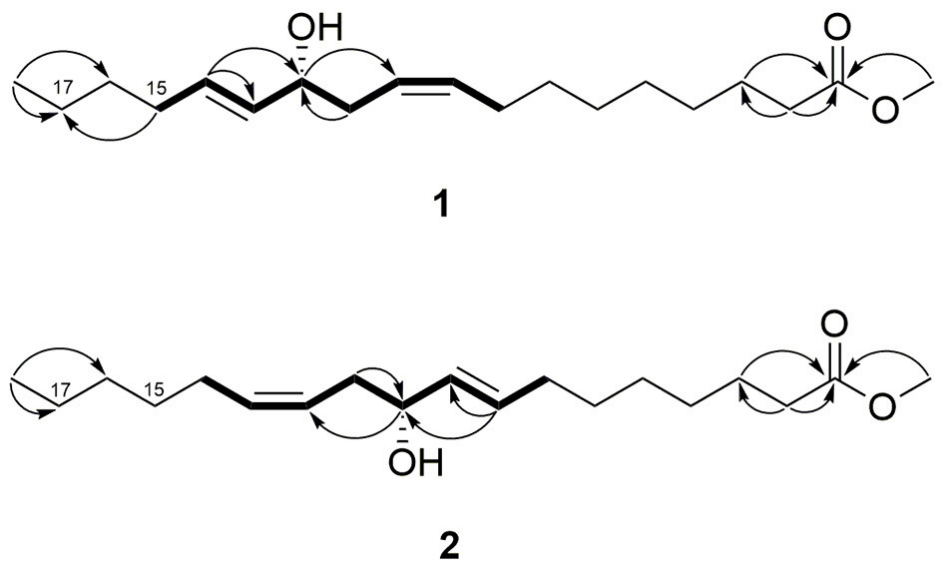
^1^H-^1^H COSY (bold bonds) and selected ^1^H-^13^C HMBC (arrows, proton to carbon) correlations of **1** and **2**.

Compound **1** was isolated as a colorless oil. The NMR data (^1^H, ^13^C, and HMQC, Table 1, Figures S1–S6) revealed the presence of two olefinic double bonds (δ_H_ 5.44, m, 3H, δ_C_ 132.6, 126.4, 133.9; δ_H_ 5.62, dt, *J* = 15.2, 6.4, δ_C_ 132.6) and an allylic hydroxy group (δ_H_ 3.99, q, *J* = 6.8 Hz) together with carbonyl group (δ_C_ 176.0), and a methoxy group (δ_H_ 3.65, s). The COSY between C-8 and C-15 together with the HMBC correlations established the 1,5-dien-4-hydroxy moiety in the long chain structure. The HMBC correlation between H-15 (δ_H_ 2.05) and C-17 (δ_C_ 23.2) suggested the position of the 1,5-dien-4-hydroxy moiety (Figure 2), which was further confirmed by GC-EIMS analysis, showing a typical fragment of *m*/*z* = 113 (C_7_H_13_O). The ^1^H NMR and GC-EIMS data were in agreement with the previously reported data of 12-hydroxy-9*Z*,13*E*-octadecadienoic acid methyl ester (Koshino et al., 1987). The stereochemistry of the hydroxy group was determined by measuring the optical rotation (${\left[\alpha \right]}_{\text{D}}^{25}$ = −257, *c* 0.06, EtOH), and comparing the result with the previously reported value (${\left[\alpha \right]}_{\text{D}}^{25}$ = −6.25, *c* 0.32, EtOH) (Koshino et al., 1987). Compound **1** was identified as 12*R*-hydroxy-9*Z*,13*E*-octadecadienoic acid methyl ester.

Compound **2** was also isolated as a colorless oil. The NMR data (^1^H, ^13^C and HMQC, Table 1, Figures S8–S13) were similar to compound **1**, indicating the presence of two olefinic double bonds (δ_H_ 5.44, m, 3H, δ_C_ 132.4, 126.4, 134.0; δ_H_ 5.62, dt, *J* = 15.2, 6.8, δ_C_ 132.7) and an allylic hydroxy group (δ_H_ 3.99, q, *J* = 6.8 Hz) in the long chain of the methylated fatty acid (δ_H_ 3.65, s). Similar COSY correlations to compound **1**, indicated the presence of similar 1,5-dien-4-hydroxy moiety. However, the absence of HMBC correlation between H-15 and C-17 indicated different position of the 1,5-dien-4-hydroxy moiety (Figure 2). The reverse position of the moiety was further elaborated based on the GC-EIMS analysis, revealing a fragment of *m*/*z* = 167. The spectroscopic data were in agreement with the reported data of previously isolated fatty acid (Koshino et al., 1987). The relative configuration of the hydroxy group was assigned as *R* by means of optical rotation (${\left[\alpha \right]}_{\text{D}}^{25}$ = −266, *c* 0.06, EtOH) compared with the reported value (${\left[\alpha \right]}_{\text{D}}^{25}$ = −6.20, *c* 1.0, EtOH) (Kashihara et al., 1989). Therefore, compound **2** was identified as 10*R*-hydroxy-8*E*,12*Z*-octadecadienoic acid methyl ester.

Compound **3** was obtained as a colorless oil. The ^1^H NMR data (Table 1) revealed the presence of a trans double bond (δ_H_ 5.44, dd, *J* = 15.2, 6.8; δ_H_ 5.62, dtd, *J* = 15.2, 6.8, 4.4 Hz) and hydroxy group (δ_H_ 4.03, q, *J* = 6.4 Hz) in long-chain fatty acid, which was methylated (δ_H_ 3.66, s). The position of the double bond and hydroxy group was assigned by GC-EIMS, which showed a strong peak at *m*/*z* 155 (C_10_H_19_O) and was identical to the reported spectroscopic data (Frankel et al., 1984; Koshino et al., 1987). The comparison of the optical rotation (${\left[\alpha \right]}_{\text{D}}^{25}$ = −386, *c* 0.01, EtOH) with the reference data (${\left[\alpha \right]}_{\text{D}}^{25}$ = −2.14, *c* 0.28, EtOH) indicated *R* configuration of the hydroxy group (Koshino et al., 1987). Thus, compound **3** was assigned as 9*R*-hydroxy-10*E*-octadecenoic acid methyl ester.

Compound **4** was isolated as another colorless oil. The ^1^H NMR data (Table 1) were similar to that of compound **3**, indicating the presence of a trans double bond (δ_H_ 5.44, dd, J = 15.2, 7.2; δ_H_ 5.63, dtd, *J* = 15.2, 6.8, 3.2 Hz) and hydroxy group (δ_H_ 4.03, q, *J* = 6.4 Hz) in the long chain of the methylated fatty acid (δ_H_ 3.66, s). The position of the double bond at C-10 and the hydroxy group at C-12 was assigned by means of GC-EIMS (*m*/*z* 227 and 195) (Frankel et al., 1984). The relative configuration of the hydroxy group was determined as *R* by means of optical rotation (${\left[\alpha \right]}_{\text{D}}^{25}$ = −80, *c* 0.015, EtOH), in correlation with the negative optical rotation of other isolated compounds. The structure of compound **4** was assigned as 12R^*^-hydroxy-10*E*-octadecenoic acid methyl ester.

Moreover, a mixture of lipids (A: palmitic acid methyl ester, stearic acid methyl ester, oleic acid methyl ester, margaric acid methyl ester, pentadecanoic acid methyl ester) and mixtures of steroids (B: β-sitost-4-en-3-one, stigmasta-4,22-dien-3-one, campest-4-en-3-one; C: β-sitosterol, stigmasterol; D: stigmasterol-3-*O*-β-D-glucoside, β-sitosterol-3-*O*-β-D-glucoside) were extracted from the rhizomes EtOAc fraction by column chromatography and identified by ^1^H NMR and GC-MS analyses (mixture A, see Figure S17; mixture B, see Figure S18). These mixtures were inactive in the cytotoxic (at 200 μg/ml), anti-inflammatory (at 10 μg/ml) or anti-allergic (up to 200 μg/ml) assays (data not shown).

The isolated hydroxylated fatty acids were obtained from *Typhonium* sp. for the first time. Moreover, ^13^C NMR data, as well as 2D NMR data of **1** and **2**, were never reported before. These fatty acids might be biosynthesized by photooxidation or enzymatic oxidation (possibly via endophytic symbiosis) of the essential unsaturated fatty acid, linoleic acid (Koshino et al., 1987), which was previously identified as one of the major components of *T. blumei* (Korinek et al., 2016a).

### Bioactivity of isolated compounds

We evaluated the anti-inflammatory and anti-allergic activities of the isolated compounds (**Tables 3**, **4**). As reported before, the methylation of fatty acids decreased their anti-inflammatory activity (Hwang et al., 2009). Compound **1**, the methyl ester of a hydroxylated fatty acid, did not exhibit anti-inflammatory activity in superoxide anion generation or elastase release assays (IC_50_ > 10 μM) (**Table 3**). Both compounds **1** and **2**, the methyl esters of hydroxylated fatty acids possessing two double bonds, showed anti-allergic inhibitory activity on A23187- and antigen-induced degranulation in RBL-2H3 cells with IC_50_ values 146.4–181.5 μM (**Table 4**). Compounds **1**–**3** were previously reported to exert fungitoxic activity against *Cladosporium herbarum*. They were related to the resistant mechanism of infected timothy plants (*Phleum pratense*) by phytopathogenic fungi (Koshino et al., 1987). Several oxygenated unsaturated fatty acids were previously described in rice plants as self-defensive substances against rice blast fungal disease (Kato et al., 1984). Thus, the isolated oxidation products of linoleic acid, compounds **1** and **2**, may contribute to the self-defensive function and bioactivity of *T. blumei*.

### *In vitro* cytotoxic, anti-inflammatory and anti-allergic activities of fatty acids and their structure-activity relationship (SAR)

The high content of saturated and unsaturated fatty acids in *T. blumei* (Korinek et al., 2016a) together with the isolation of hydroxylated fatty acids (**1**–**4**) motivated us to further study the bioactivity of a series of saturated, unsaturated and hydroxylated fatty acids. Although several fatty acids were reported to exert anti-allergic (Willemsen, 2016), anti-inflammatory (Hwang et al., 2009; Calder, 2015), and cytotoxic effects (Jing et al., 2013; Vangaveti et al., 2016), complex bioactivity data describing the structural requirements of fatty acids for the bioactivities are lacking. Moreover, very few reports examined the bioactivity of hydroxylated fatty acids (Hou, 2009).

We obtained a series of saturated, unsaturated and hydroxylated fatty acids (Figure 1) and evaluated their *in vitro* cytotoxic, anti-inflammatory and anti-allergic activities with the aim to elucidate the possible correlation between the activity and certain structural features, including level and position of unsaturation, hydroxylation, position of the hydroxy group or acid methylated derivatives.

#### Cytotoxicity

Cytotoxic activity against hepatoma (HepG2), breast (MDA-MB-231) or lung (A549) cancer cell lines was exerted by γ-linolenic acid (C18:3n-6), DHA (C22:6n-3), 12-hydroxystearic acid (C18:0, 12-OH), and ricinoleic acid (C18:1n-9, cis, 12-OH) without any selective toxicity to a specific cell line (Table 2). 12-Hydroxystearic acid exerted the most potent cytotoxic activity among the tested compounds (IC_50_ 86.3–119.0 μM for three cell lines). Previous literature demonstrated *in vitro* cytotoxic effects of fatty acids (such as DHA) and lipids on several cancer cell lines, inducing apoptosis by modulation of various signaling molecules and pathways (Fauser et al., 2011; Hsu et al., 2011).

**Table 2:** Cytotoxic data of fatty acids.

			**Cytotoxicity assay^a^**		
**Name**	**Abbreviation**	**Mol. weight**	**HepG2^b^ IC_50_ (μM)^c^**	**MDA-MB-231^b^ IC_50_ (μM)^c^**	**A549^b^ IC_50_ (μM)^c^**
Palmitic acid	C16:0	256.4	NS	NS	NS
Palmitic acid methyl ester	C16:0, me	270.5	NS	NS	NS
Stearic acid	C18:0	284.5	NS	NS	NS
Stearic acid methyl ester	C18:0, me	298.5	NS	NS	NS
Undecylenic acid	C11:1n-1	184.3	NS	NS	NS
Undecylenic acid methyl ester	C11:1n-1, me	198.3	NS	NS	NS
*cis*-Vaccenic acid	C18:1n-7	282.5	NS	NS	NS
*cis*-Vaccenic acid methyl ester	C18:1n-7, me	296.5	NS	NS	NS
Oleic acid	C18:1n-9	282.5	NS	NS	NS
Oleic acid methyl ester	C18:1n-9, me	296.5	NS	NS	NS
Petroselinic acid	C18:1n-12	282.5	NS	NS	NS
Petroselinic acid methyl ester	C18:1n-12, me	296.5	NS	NS	NS
Linoleic acid	C18:2n-6	280.5	NS	NS	NS
Linoleic acid methyl ester	C18:2n-6, me	294.5	NS	NS	NS
Conjugated (9*Z*,11*E*)-linoleic acid	C18:2n-7	280.5	NS	NS	NS
α-Linolenic acid	C18:3n-3	278.4	NS	NS	NS
α-Linolenic acid methyl ester	C18:3n-3, me	292.5	NS	NS	NS
γ-Linolenic acid	C18:3n-6	278.4	>200^d^	192.7	190.9
*cis*-5,8,11,14,17-Eicosapentaenoic acid (EPA)	C20:5n-3	302.5	>200^d^	>200^d^	NS
*cis*-4,7,10,13,16,19-Docosahexaenoic acid (DHA)	C22:6n-3	328.5	146.9	140.0	>200^d^
2-Hydroxystearic acid	C18:0, 2-OH	300.5	NS	NS	NS
12-Hydroxystearic acid	C18:0, 12-OH	300.5	119.0	105.0	86.3
12-Hydroxystearic acid methyl ester	C18:0, 12-OH, me	314.5	NS	NS	NS
12-Oxostearic acid methyl ester	C18:0, 12-oxo, me	312.5	NS	NS	NS
2-Hydroxy-9*Z*-octadecenoic acid (minerval, 2OHOA)	C18:1n-9, *cis*, 2-OH	298.5	NS	NS	NS
12*R*-Hydroxy-9*Z*-octadecenoic acid (ricinoleic acid)	C18:1n-9, *cis*, 12-OH	298.5	174.8	NS	154.8
12*R*-Hydroxy-9*Z*-octadecenoic acid (ricinoleic acid) methyl ester	C18:1n-9, *cis*, 12-OH, me	312.5	NS	NS	NS
12*R*-Hydroxy-9*E*-octadecenoic acid (ricinelaidic acid)	C18:1n-9, *trans*, 12-OH	298.5	NS	NS	NS
Doxorubicin			0.62	0.67	0.90

a *The cytotoxicity was evaluated using MTT viability assay; IC_50_ values; results are presented as mean (n = 3); NS, not significant at 200 μM (n = 1)*.

b *Hep-G2, Human hepatocellular carcinoma cells; MDA-MB-231, human breast adenocarcinoma cells; A549, human lung adenocarcinoma cells*.

c *IC_50_ values express the concentration of the sample required to inhibit cancer cell growth by 50%*.

d *>200 μM means that the sample inhibited the growth of the cells, but didn't reach IC_50_ (n = 3)*.

#### Anti-inflammatory activity

Following up on the potent anti-inflammatory activity of a series of saturated and unsaturated fatty acids previously reported by our group (Hwang et al., 2009), we aimed to further extend the list of the tested fatty acids, particularly focusing on those containing a hydroxy group. In agreement with previous data, all tested methyl esters were inactive (Table 3). Similarly, free acidic group and unsaturation were the structural requirements for the anti-inflammatory activity of the tested fatty acids. Petroselinic acid (C18:1n-12), conjugated linoleic acid (C18:2n-7), DHA (C22:6n-3), and EPA (C20:5n-3) exerted significant anti-inflammatory activity by inhibiting superoxide anion generation (IC_50_ 2.08–3.73 μM) and elastase release (IC_50_ 1.26–2.54 μM), which were comparable to the previously reported data for oleic, *cis*-vaccinic, linoleic, and α-linolenic acids (Hwang et al., 2009). Interestingly, the lack of activity of undecylenic acid (C11:1n-1) indicated that short chain FA did not exert activity. In agreement with our previous results (Hwang et al., 2009), plain saturated fatty acids (palmitic acid, C16:0, and stearic acid, C18:0) were inactive, and similarly, 2-OH or 12-OH saturated fatty acids did not show anti-inflammatory activity. On the other hand, the hydroxylated monounsaturated fatty acids, 2-hydroxy-9Z-octadecenoic acid (2OHOA, C18:1n-9, cis, 2-OH), and ricinelaidic acid (C18:1n-9, trans, 12-OH) exerted significant inhibitory activity on both superoxide anion generation (IC_50_ 2.40 and 2.32 μM, respectively) and elastase release (IC_50_ 2.02 and 1.63 μM, respectively) by human neutrophils. The comparison of the superoxide generation and elastase release inhibitory activities of the hydroxylated monounsaturated fatty acids with the monounsaturated fatty acids without a hydroxy group (oleic acid IC_50_ 2.56 and 1.40 μM, respectively (Hwang et al., 2009), and petroselinic acid IC_50_ 2.14 and 1.39 μM, respectively), showed that the addition of a hydroxy group yielded similar activity.

**Table 3:** Anti-inflammatory data of fatty acids.

		**Anti-inflammatory assay^a^**	
		**Superoxide anion**	**Elastase release**
**Name**	**Abbreviation**	**IC_50_ (μM)^b^**	**IC_50_ (μM)^b^**
Palmitic acid	C16:0	>10	>10
Palmitic acid methyl ester	C16:0, me	>10	>10
Stearic acid	C18:0	>10^c^	>10^c^
Stearic acid methyl ester	C18:0, me	>10	>10
Undecylenic acid	C11:1n-1	>10	>10
Undecylenic acid methyl ester	C11:1n-1, me	>10	>10
*cis*-Vaccenic acid	C18:1n-7	2.31 ± 0.20^c^	1.36 ± 0.07^c^
*cis*-Vaccenic acid methyl ester	C18:1n-7, me	>10	>10
Oleic acid	C18:1n-9	2.56 ± 0.13^c^	1.40 ± 0.07^c^
Oleic acid methyl ester	C18:1n-9, me	>10^c^	>10^c^
Petroselinic acid	C18:1n-12	2.14 ± 0.47	1.39 ± 0.07
Petroselinic acid methyl ester	C18:1n-12, me	>10	>10
Linoleic acid	C18:2n-6	2.64 ± 0.17^c^	1.80 ± 0.12^c^
Linoleic acid methyl ester	C18:2n-6, me	>10	>10
Conjugated (9*Z*,11*E*)-linoleic acid	C18:2n-7	2.08 ± 0.50	1.26 ± 0.15
α-Linolenic acid	C18:3n-3	3.49 ± 0.22	4.57 ± 0.08
α-Linolenic acid methyl ester	C18:3n-3, me	>10	>10
γ-Linolenic acid	C18:3n-6	4.57 ± 0.25^c^	3.17 ± 0.18^c^
*cis*-5,8,11,14,17-Eicosapentaenoic acid (EPA)	C20:5n-3	3.73 ± 0.91	1.52 ± 0.14
*cis*-4,7,10,13,16,19-Docosahexaenoic acid (DHA)	C22:6n-3	2.82 ± 0.44	2.54 ± 0.13
2-Hydroxystearic acid	C18:0, 2-OH	>10	>10
12-Hydroxystearic acid	C18:0, 12-OH	>10	>10
12-Hydroxystearic acid methyl ester	C18:0, 12-OH, me	>10	>10
12-Oxostearic acid methyl ester	C18:0, 12-oxo, me	>10	>10
2-Hydroxy-9*Z*-octadecenoic acid (minerval, 2OHOA)	C18:1n-9, *cis*, 2-OH	2.40 ± 0.69	2.02 ± 0.70
12*R*-Hydroxy-9*Z*-octadecenoic acid (ricinoleic acid)	C18:1n-9, *cis*, 12-OH	>10^d^	>10^d^
12*R*-Hydroxy-9*Z*-octadecenoic acid (ricinoleic acid) methyl ester	C18:1n-9, *cis*, 12-OH, me	>10	Enhancing effect^e^
12*R*-Hydroxy-9*E*-octadecenoic acid (ricinelaidic acid)	C18:1n-9, *trans*, 12-OH	2.32 ± 0.59	1.63 ± 0.16
12*R*-Hydroxy-9*Z*,13*E*-octadecadienoic acid methyl ester (**1**)	C18:2n-5, 12-OH, me	>10	>10
Genistein		1.37 ± 0.53	40.0 ± 8.9

a *Anti-inflammatory capacity was evaluated by superoxide anion generation and elastase release assays in human neutrophils using fMLF/CB as an inducer. IC_50_ values; results are presented as mean ± SEM (n = 3–4); compared with the control value (fMLF/CB)*.

b *IC_50_ values express the concentration of the sample required to inhibit superoxide anion generation or elastase release by 50%*.

c *The samples were tested in Hwang et al. (2009)*.

d *Ricinoleic acid exerted significant inhibitory activity in superoxide anion generation (39.9 ± 5.6%) and elastase release (39.9 ± 4.1%) assay at 10 μM*.

e *The compound showed enhancing effects on elastase release at 10 μM (42.8 ± 6.5%) in the presence of CB. Compared with fMLF/CB (as 100%)*.

#### Anti-allergic activity

A23187- and antigen-induced degranulation assays by mast cells (RBL-2H3) were utilized to evaluate the anti-allergic activity of all fatty acids (Table 4). The high doses used of fatty acids in the assay may be justified by the fact that human beings consume large amounts of these compounds on regular basis (Sun et al., 2015). Potential cytotoxic effect toward RBL-2H3 cells was measured by MTT viability assay and only non-toxic concentrations (viability over 85%, Table S1) were further used in the degranulation assay. Among the tested compounds, none of the unsaturated fatty acids, regardless of the double bond position, showed inhibitory activity on A23187- or antigen-induced degranulation by mast cells. Interestingly, at 1,000 μM the plain saturated palmitic (C16:0) and stearic (C18:0) fatty acids, common in plants, particularly in seeds and oils, exerted significant inhibition on A23187-induced (29.7 ± 4.7 and 35.3 ± 2.3%, respectively) and antigen-induced (40.7 ± 3.8 and 45.3 ± 1.2%, respectively) degranulation. This finding may be of interest to companies focusing on the preparation of oils and food supplements. Also, the hydroxylated saturated fatty acids exerted anti-allergic activity, as 12-hydroxystearic acid (C18:0, 12-OH, IC_50_ 162.9 μM) and 12-hydroxystearic acid methyl ester (C18:0, 12-OH, me, IC_50_ 491.2 μM) in A23187-induced degranulation assay. Monounsaturated hydroxylated fatty acids (2OHOA, C18:1n-9, cis, 2-OH and ricinoleic acid methyl ester C18:1n-9, cis, 12-OH, me) at 1000 μM showed significant inhibitory effect on A23187-induced (20.0 ± 4.4 and 43.0 ± 2.6%, respectively) and antigen-induced (20.3 ± 3.1, and 58.0 ± 2.6%, respectively) degranulation. Among the tested hydroxylated unsaturated fatty acids, the hydroxylated diunsaturated 9*S*-hydroxy-10*E*,12*Z*-octadecadienoic acid (9(S)-HODE, C18:2n-6, 9-OH, IC_50_ 92.4 and 49.7 μM in A23187- and antigen-induced degranulation assay, respectively) showed a more potent anti-allergic effect in comparison with the monounsaturated hydroxylated fatty acids. In correlation with this result, the isolated hydroxylated diunsaturated compounds **1** and **2** also exerted significant anti-allergic activities, with IC_50_ values 146.4–181.5 μM.

**Table 4:** Anti-allergic data of fatty acids.

		**Anti-allergic assay^a^**	
		**A23187-induced**	**Antigen-induced**
**Name**	**Abbreviation**	**IC_50_ (μM)^b^**	**IC_50_ (μM)^b^**
Palmitic acid	C16:0	>1,000^c^	>1,000^c^
Palmitic acid methyl ester	C16:0, me	>1,000	>1,000
Stearic acid	C18:0	>1,000^d^	>1,000^d^
Stearic acid methyl ester	C18:0, me	>1,000	>1,000
Undecylenic acid	C11:1n-1	>500^e^	>500^e^
Undecylenic acid methyl ester	C11:1n-1, me	>1,000	>1,000
*cis*-Vaccenic acid	C18:1n-7	>500^e^	>500^e^
*cis*-Vaccenic acid methyl ester	C18:1n-7, me	>1,000	>1,000
Oleic acid	C18:1n-9	>500^e^	>500^e^
Oleic acid methyl ester	C18:1n-9, me	>1,000	>1,000
Petroselinic acid	C18:1n-12	>500^e^	>500^e^
Petroselinic acid methyl ester	C18:1n-12, me	>1,000	>1,000
Linoleic acid	C18:2n-6	>500^e^	>500^e^
Linoleic acid methyl ester	C18:2n-6, me	>500^e^	>500^e^
Conjugated (9*Z*,11*E*)-linoleic acid	C18:2n-7	>200^f^	>200^f^
α-Linolenic acid	C18:3n-3	>200^f^	>200^f^
α-Linolenic acid methyl ester	C18:3n-3, me	>500^e^	>500^e^
γ-Linolenic acid	C18:3n-6	>200^f^	>200^f^
*cis*-5,8,11,14,17-Eicosapentaenoic acid (EPA)	C20:5n-3	>200^f^	>200^f^
*cis*-4,7,10,13,16,19-Docosahexaenoic acid (DHA)	C22:6n-3	>200^f^	>200^f^
2-Hydroxystearic acid	C18:0, 2-OH	>200^g^	>200^g^
12-Hydroxystearic acid	C18:0, 12-OH	162.9^g^	>200^g^
12-Hydroxystearic acid methyl ester	C18:0, 12-OH, me	491.2	>1,000
12-Oxostearic acid methyl ester	C18:0, 12-oxo, me	>1,000^h^	>1,000^h^
2-Hydroxy-9*Z*-octadecenoic acid (minerval, 2OHOA)	C18:1n-9, *cis*, 2-OH	>1,000^i^	>1,000^i^
12*R*-Hydroxy-9*Z*-octadecenoic acid (ricinoleic acid)	C18:1n-9, *cis*, 12-OH	>200^f^	>200^f^
12*R*-Hydroxy-9*Z*-octadecenoic acid (ricinoleic acid) methyl ester	C18:1n-9, *cis*, 12-OH, me	>1,000^j^	913.0
12*R*-Hydroxy-9*E*-octadecenoic acid (ricinelaidic acid)	C18:1n-9, *trans*, 12-OH	>500^e^	>500^e^
12*R*-Hydroxy-9*Z*,13*E*-octadecadienoic acid methyl ester (**1**)	C18:2n-5, 12-OH, me	146.4^f^	153.0^f^
9*S*-Hydroxy-10*E*,12*Z*-octadecadienoic acid (9(S)-HODE)	C18:2n-6, 9-OH	92.4^k^	49.7^k^
10*R*-Hydroxy-8*E*,12*Z*-octadecadienoic acid methyl ester (**2**)	C18:2n-6, 10-OH, me	181.5^f^	154.1^f^
Dexamethasone		80% at 10 nM	62% at 10 nM

a *The inhibition of degranulation was assessed by A23187- or antigen-induced β-hexosaminidase release assays in RBL-2H3 cells; IC_50_ values; results are presented as mean (n = 3); compared with the control value (A23187 or antigen). For detail information about cytotoxicity toward RBL-2H3 cells and degranulation inhibitory data (1μM–1,000 μM) please refer to Table S1 in Supporting information*.

b *IC_50_ values express the concentration of the sample required to inhibit degranulation by 50%*.

c *Palmitic acid exerted significant inhibitory activity in both A23187- (29.7 ± 4.7%) and antigen-induced (40.7 ± 3.8%) degranulation assay at 1,000 μM*.

d *Stearic acid exerted significant inhibitory activity in both A23187- (35.3 ± 2.3%) and antigen-induced (45.3 ± 1.2%) degranulation assay at 1,000 μM*.

e *Sample exerted cytotoxic effects (viability <80%) toward RBL-2H3 cells at concentrations higher than 500 μM*.

f *Sample exerted cytotoxic effects (viability <80%) toward RBL-2H3 cells at concentrations higher than 200 μM*.

g *Micelles were formed upon addition into the medium at concentrations above 200 μM, therefore the results at these concentrations couldn't be justified*.

h *12-Oxostearic acid methyl ester exerted significant inhibitory activity in both A23187- (40.3 ± 3.5%) and antigen-induced (23.7 ± 3.1%) degranulation assay at 1,000 μM*.

i *Minerval (2OHOA) exerted significant inhibitory activity in both A23187- (20.0 ± 4.4%) and antigen-induced (20.3 ± 3.1%) degranulation assay at 1,000 μM*.

j *Ricinoleic acid methyl ester exerted significant inhibitory activity in A23187-induced (43.0 ± 2.6%) degranulation assay at 1,000 μM*.

k *The highest tested concentration of 9(S)HODE was 100 μM*.

In comparison with a series of unsaturated fatty acids (including oleic acid, linoleic acid, linolenic acid, DHA, and EPA), only hydroxylated fatty acids exerted anti-allergic activity. Based on our results, in contrast with the anti-inflammatory effect, a free acid is not a requirement for the anti-allergic activity of fatty acids but may influence their cytotoxicity (as demonstrated by ricinoleic acid vs. its methyl ester), and the level of inhibition of degranulation (palmitic acid vs. its methyl ester). Furthermore, the substitution of the hydroxy group with a keto group resulted in weaker anti-allergic activities. 12-Hydroxystearic acid (C18:0, 12-OH) or its methyl ester (C18:0, 12-OH, me) exhibited more potent anti-allergic activity compared with 12-oxostearic acid methyl ester (C18:0, 12-oxo, me).

DHA and EPA (n-3) fatty acids at 100 μM were previously reported to exert anti-allergic effects in phorbol 12-myristate 13-acetate- and ionomycin-activated RBL-2H3 mast cells (Jin et al., 2014; Willemsen, 2016). However, in our experimental model using A23187 or antigen as inducers, DHA and EPA did not show significant inhibitory activity on degranulation in mast cells at a non-toxic concentration of 200 μM.

### ChemGPS, physico-chemical properties of fatty acids

**Figure 3:**
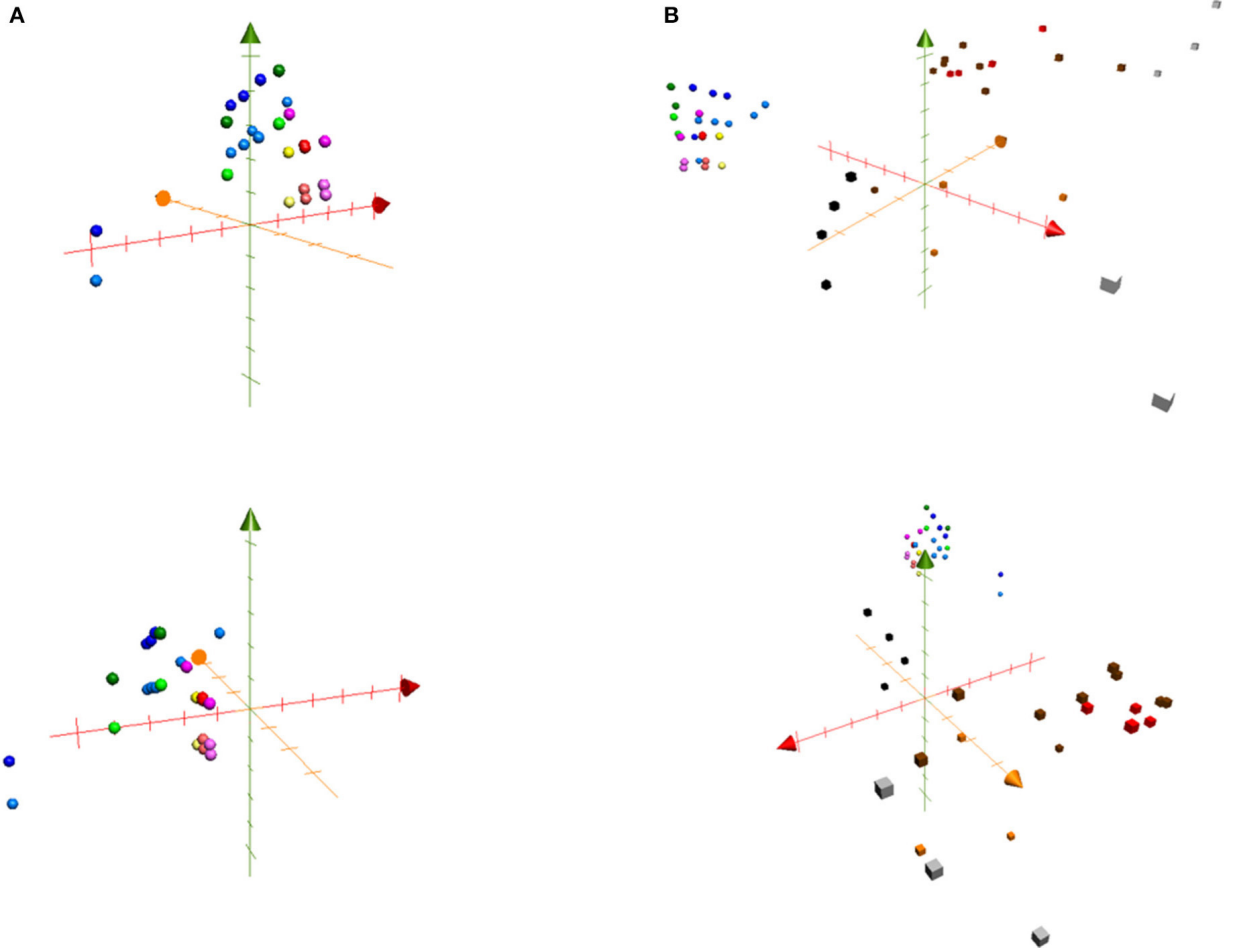
ChemGPS-NP analysis of long-chain fatty acids. The isolated fatty acids from *Typhonium blumei* together with series of saturated, unsaturated and oxygenated fatty acids were plotted into the three dimensions consisting of PC1 (principal components 1; red; represents size, shape, and polarizability), PC2 (orange; aromatic- and conjugation-related properties), and PC3 (green; lipophilicity, polarity, and H-bond capacity). **(A)** The plots represent saturated fatty acids (blue dots), unsaturated fatty acids (green dots), saturated oxygenated fatty acids (purple dots), monounsaturated hydroxy fatty acids (red dots) and diunsaturated hydroxy fatty acids (yellow dots). Acids are shown in a light tone, while methyl esters in dark tone of the corresponding color. Diunsaturated fatty acids and methyl esters (yellow dots) were found to exert the best anti-allergic activity (**1, 2**, 9(S)-HODE). **(B)** The following clinically used anti-allergic drugs were plotted into chemical space: glucocorticoids (black squares), immunosuppressants (white squares), leukotriene inhibitors (gray squares), mast cell stabilizers (orange squares), antihistaminics (brown squares) and “dual antihistaminics” known to inhibit mast cell degranulation (red squares).

ChemGPS-NP (chemical global positioning system for natural products) was developed as a computational model based on the principal component analysis (PCA) of physico-chemical properties of nature-derived compounds. Compounds are placed into a chemical space based on their properties, which can be calculated from simplified molecular input line entry specification (SMILES) structure data, followed by execution of score prediction in ChemGPS-NP. The system provides a bioinformatics tool for plotting and navigating biologically relevant chemical spaces (Larsson et al., 2007; Lai et al., 2016). It can further serve as a tool for discussing the structure-activity relationships as well as to predict possible bioactivities for specific groups of compounds. We plotted the long-chain fatty acids into ChemGPS-NP (http://chemgps.bmc.uu.se) with the aim to observe a possible correlation between oxygenation and bioactivity in relevant chemical space (Figure 3). Anti-allergic hydroxylated fatty acids and their methyl esters (purple, red and yellow dots), explored particularly by the first dimension (PC1, red axis, representing size, shape, and polarizability), formed a cluster in the analyzing graphic (Figure 3A). Thus, fatty acids possessing hydroxy groups which would be plotted in the space toward an increase in size, shape, and polarizability (PC1, red axis) may yield better anti-allergic activity.

Furthermore, we plotted clinical anti-allergic drugs, namely glucocorticoids (black squares), immunosuppressants (white squares), leukotriene inhibitors (gray squares), mast cells stabilizers (orange squares), and antihistaminics (brown and red squares), into the chemical space (PC1, PC2, PC3). Antihistaminics analyzed, including antihistaminics of 1st and 2nd generation, were further divided into two groups: antihistaminics with regular histamine receptor antagonizing capacity (brown squares) and antihistaminics with a dual mode of action that are known to additionally inhibit mast cell degranulation (“dual antihistaminics,” red squares). The drugs formed different clusters distant from fatty acids group (Figure 3B). Among the different groups, the antihistaminics (brown squares) and “dual antihistaminics” known to inhibit mast cell degranulation (red squares), shared chemical properties regarding the first dimension (PC1, red axis, representing size, shape, and polarizability) and the third dimension (PC3, green axis, representing lipophilicity, polarity, and H-bond capacity) compared with the fatty acids group. Taken together, the anti-allergic fatty acids group represents a new cluster regarding the anti-allergic activity, showing a new pattern of chemical properties that could be of use for the future development of anti-allergic drugs.

## Discussion

12/15-Lipoxygenase (12/15-LOX) catalyzes the oxidation of free and esterified fatty acids, thus forming a variety of bioactive lipid mediators. 12/15-LOX plays a key role in the regulation of different homeostatic processes as well as in the pathogenesis of several diseases (Uderhardt and Kronke, 2012; Lopez-Vicario et al., 2016). Oxygenated products of unsaturated fatty acids possess immune modulatory properties that effect inflammatory responses (Duvall and Levy, 2016; Whelan et al., 2016). Several studies investigated the properties and function of fatty acid derivatives, particularly arachidonic acid (AA, C20:4n-6) and DHA (C22:6n-3) and their related metabolic pathways (Lopez-Vicario et al., 2016). Elegant reviews described the pro-inflammatory as well as the inflammatory-resolving functions of various 12/15-LOX products (Kuhn and O'Donnell, 2006; Ackermann et al., 2017). For example, 15S-hydroxy-5Z,8Z,11Z,13E-eicosatetraenoic acid (15(S)-HETE), but not its methyl ester, was reported to serve as a mediator limiting and/or reversing acute inflammation by exhibiting counterbalancing effects on neutrophil trafficking across endothelium. 15(S)-HETE was found to inhibit respiratory burst and degranulation of neutrophils activated with receptor-specific agonists, such as fMLF, platelet-activating factor, and leukotriene B4 (Smith et al., 1993; Takata et al., 1994). In agreement with the anti-inflammatory effect of 15(S)-HETE, our findings revealed that unsaturated hydroxylated free fatty acids (2-hydroxy-9Z-octadecenoic acid, 12R-hydroxy-9E-octadecenoic acid) inhibited fMLF/CB-induced superoxide generation and elastase release in human neutrophils (Table 3). The introduction of either hydroperoxy or hydroxyl groups into arachidonic acid (AA, 20:4n-6) abolished its neutrophil adherence stimulating ability and decreased its capacity to stimulate the release of both specific and azurophilic granules (Bates et al., 1995). It was found that the conversion of AA to 15S-hydroperoxy-5Z,8Z,11Z,13E-eicosatetraenoic acid (15-HPETE) led to the loss of the compound ability to activate the neutrophil responses of superoxide production, degranulation, and adherence (Huang et al., 1997; Ferrante and Ferrante, 2005). Moreover, 15-HPETE suppressed the lipopolysaccharide (LPS)-induced production and mRNA expression of tumor necrosis factor alpha (TNF-α), and significantly reduced the LPS-induced translocation of protein kinase C (PKC) (Ferrante et al., 1997). Oxidative products of 12/15-LOX were, moreover, reported as coactivators of anti-inflammatory peroxisome proliferator-activated receptor (PPARγ) (Huang et al., 1999), regulators of cytokine generation, and modulators of gene expression related to inflammation resolution (Kuhn and O'Donnell, 2006).

During the innate and adaptive immune response, 12/15-LOX and its products exert both pro- and anti-inflammatory effects (Ackermann et al., 2017). Recently, some of the oxygenated fatty acid metabolites of n-3 unsaturated fatty acids, such as 17,18-epoxy-5Z,8Z,11Z,14Z-eicosatetraenoic and 17,18-dihydroxy-5Z,8Z,11Z,14Z-eicosatetraenoic acid, demonstrated the anti-allergic effect *in vivo* due to impairment of mast cells degranulation (Kunisawa et al., 2015). Similarly, according to our current findings, oxygenated fatty acids (**1, 2**, 9(S)-HODE) formed upon the oxidation of linoleic acid exerted suppressive effects on the mast cells degranulation (Table 4). It has been reported that the high intake of unsaturated fatty acids in pregnancy or early childhood may prevent the development of allergic diseases, particularly asthma and atopic eczema (Lumia et al., 2011; Willemsen, 2016). In correlation with our findings, the elucidation of whether such effects might be a consequence of oxidative metabolism of the consumed fatty acids would be warranted.

## Conclusion

According to our results, the presence of a hydroxy group in the long chain did not influence the potent anti-inflammatory activity of free unsaturated acids. Nevertheless, hydroxylation of fatty acids (or their methyl esters) seems to be a key factor for the anti-allergic activity observed in the current study (Graphical Abstract). Considering the high content in plants and high daily intake of fatty acids from food and oils, the presented results may shed light on the utilization of fatty acids and their hydroxylated products in the treatment of inflammatory and allergic diseases. Thus, the bioactivity of *T. blumei*, which is historically utilized in folk medicine, might be related to the content of fatty acids and their metabolites.

**Graphical Abstract:**
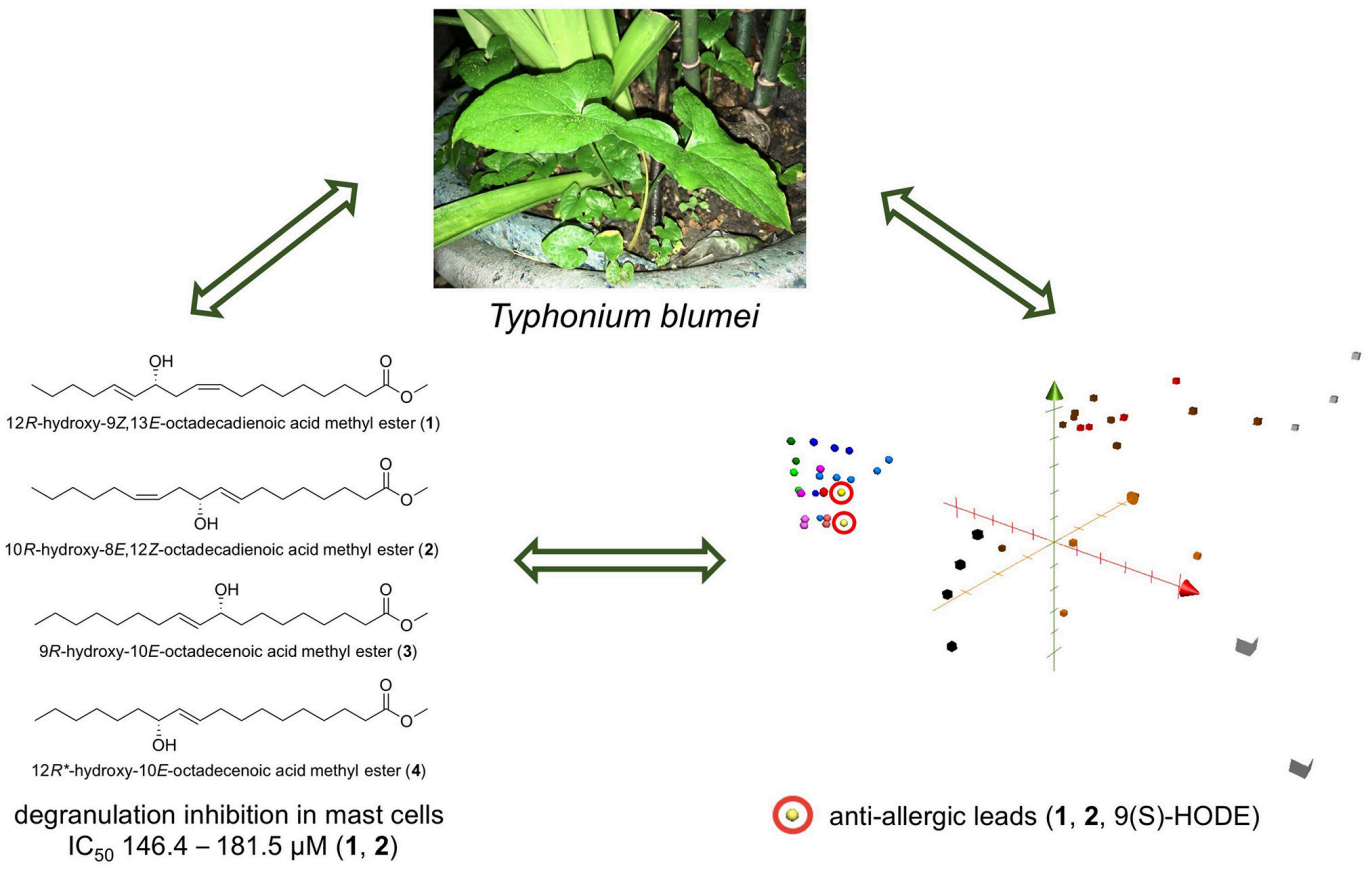
A summary of the study. Anti-allergic hydroxy fatty acids from *Typhonium blumei* may serve as lead candidates for further anti-allergic treatment development.

## Author contributions

TH, BC, and FC conceived and designed the experiments and contributed to the manuscript preparation. MK executed the phytochemical and anti-allergic experiments, analyzed the data and wrote the manuscript. SC performed cytotoxicity experiments. TH completed anti-inflammatory *in vitro* study. YT performed GC-MS experiments. KL and AB assisted with utilizing ChemGPS-NP in the study. SW, WL, and TW assisted with the phytochemical experiments. MK, ME, YC, and YW revised the manuscript. All the authors approved the final version of the manuscript.

### Conflict of interest statement

The authors declare that the research was conducted in the absence of any commercial or financial relationships that could be construed as a potential conflict of interest.

## Abbreviations

TB: *Typhonium blumei*
DHA: cis-4,7,10,13,16,19-docosahexaenoic acid
EPA: cis-5,8,11,14,17-eicosapentaenoic acid
9(S)-HODE: 9S-hydroxy-10E,12Z-octadecadienoic acid
2OHOA: 2-hydroxy-9Z-octadecenoic acid
RBL: rat basophilic leukemia
fMLF/CB: formyl-methionyl-leucyl-phenylalanine/cytochalasin B
12/15-LOX: 12/15-lipoxygenase
15-HPETE: 15S-hydroperoxy-5Z,8Z,11Z,13E-eicosatetraenoic acid
15(S)-HETE: 15S-hydroxy-5Z,8Z,11Z,13E-eicosatetraenoic acid.
