# Supplementary material for: Anti-allergic Hydroxy Fatty Acids from Typhonium blumei Explored through ChemGPS-NP
Source: Front Pharmacol. 2017 Jun 19;8:356. doi: 10.3389/fphar.2017.00356 (PMC5474496; doi:10.3389/fphar.2017.00356)
Supplement: Supplementary file 2 [file DataSheet1.pdf]

Supplementary Material

Anti-allergic Hydroxy Fatty Acids from *Typhonium blumei* Explored Through ChemGPS-NP

Michal Korinek, Yi-Hong Tsai, Mohamed El-Shazly, Kuei-Hung Lai, Anders Backlund, Shou-Fang Wu, Wan-Chun Lai, Tung-Ying Wu, Shu-Li Chen, Yang-Chang Wu, Yuan-Bin Cheng, Tsong-Long Hwang*, Bing-Hung Chen*, Fang-Rong Chang*

*** Correspondence:** T.-L. Hwang: htl@mail.cgu.edu.tw, B.-H. Chen: bhchen@kmu.edu.tw, F. R. Chang: aaronfrc@kmu.edu.tw

Content

Anti-allergic data with inhibition of degranulation data of fatty acids (1 – 1000 μM), Table S1 (attached as xlsx. file).

^{1}H NMR, ^{13}C NMR, 2D NMR data of **1** and **2** and GC-MS data of mixtures A and B (see below).

Bioactivity screening data of *T. blumei* extract and fractions (see below).

Table of Contents

Figure S1. ^{1}H NMR spectrum of compound **1** in CD_3OD, 400 MHz S3

Figure S2. ^{13}C NMR spectrum of compound **1** in CD_3OD, 100 MHz S4

Figure S3. ^{1}H-^{1}H COSY spectrum of compound **1** in CD_3OD S5

Figure S4. HSQC spectrum of compound **1** in CD_3OD S6

Figure S5. HMBC spectrum of compound **1** in CD_3OD S7

Figure S6. NOESY spectrum of compound **1** in CD_3OD S8

Figure S7. GC-EIMS spectrum of compound **1** S9

Figure S8. ^{1}H NMR spectrum of compound **2** in CD_3OD, 400 MHz S10

Figure S9. ^{13}C NMR spectrum of compound **2** in CD_3OD, 100 MHz S11

Figure S10. ^1H-^1H COSY spectrum of compound **2** in CD_3OD S12

Figure S11. HMQC spectrum of compound **2** in CD_3OD S13

Figure S12. HMBC spectrum of compound **2** in CD_3OD S14

Figure S13. NOESY spectrum of compound **2** in CD_3OD S15

Figure S14. GC-EIMS spectrum of compound **2** S16

Figure S15. GC-EIMS spectrum of compound **3** S17

Figure S16. GC-EIMS spectrum of compound **4** S18

Figure S17. GC-EIMS spectrum of mixture A S19

Figure S18. GC-EIMS spectrum of mixture B S20

Table S1. Anti-allergic data of fatty acids (1 − 1000 μM) .... see attached xlsx. file

Table S2. Anti-platelet activity of *Typhonium blumei* S21

Table S3. Cytotoxicity of *T. blumei* S22

Table S4. Anti-inflammatory activity of *T. blumei* S23

Table S5. Cytotoxicity of *T. blumei* subfractions S24

Table S6. Anti-inflammatory activity of *T. blumei* subfractions S25

Table S7. Anti-allergic activity of *T. blumei* subfractions S26

Figure S1. ^{1}H NMR spectrum of compound **1** in CD_3OD, 400 MHz

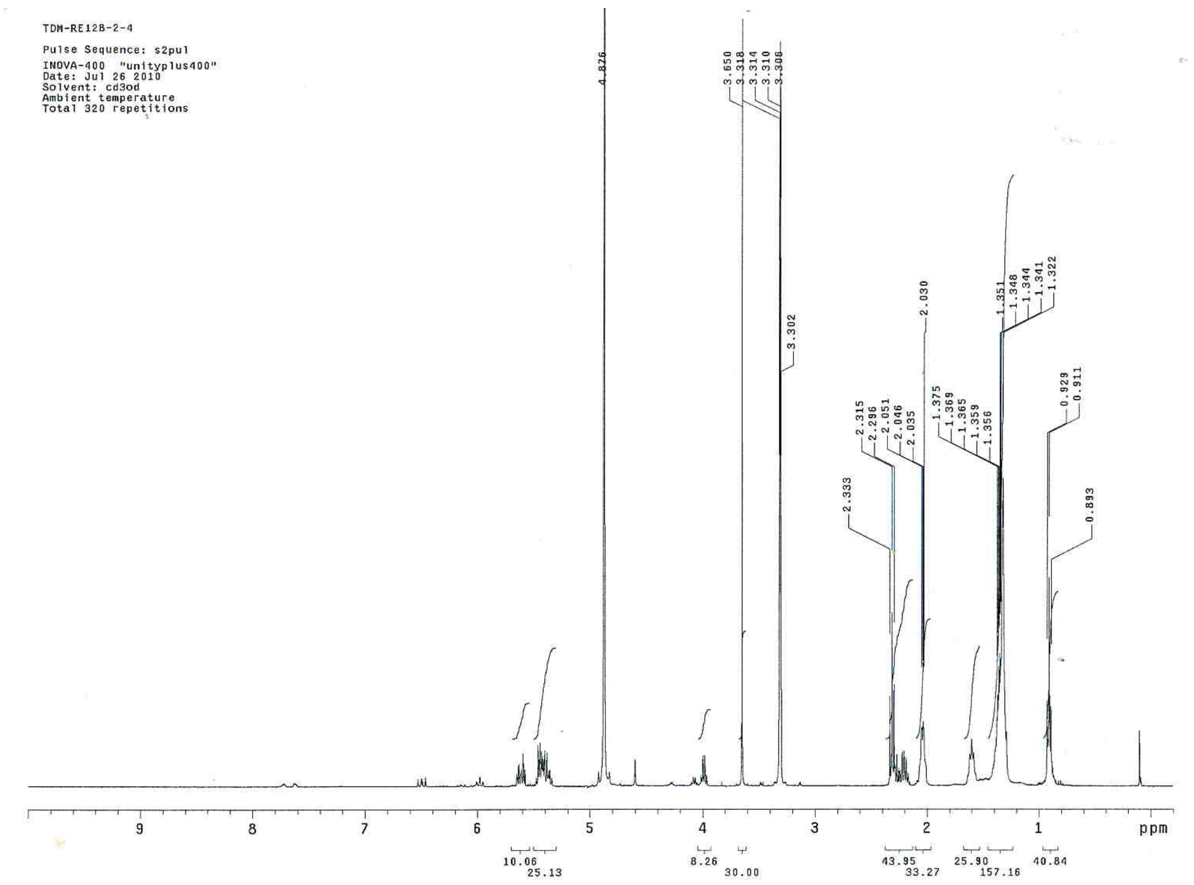

Figure S2. ^{13}C NMR spectrum of compound **1** in CD_3OD, 100 MHz

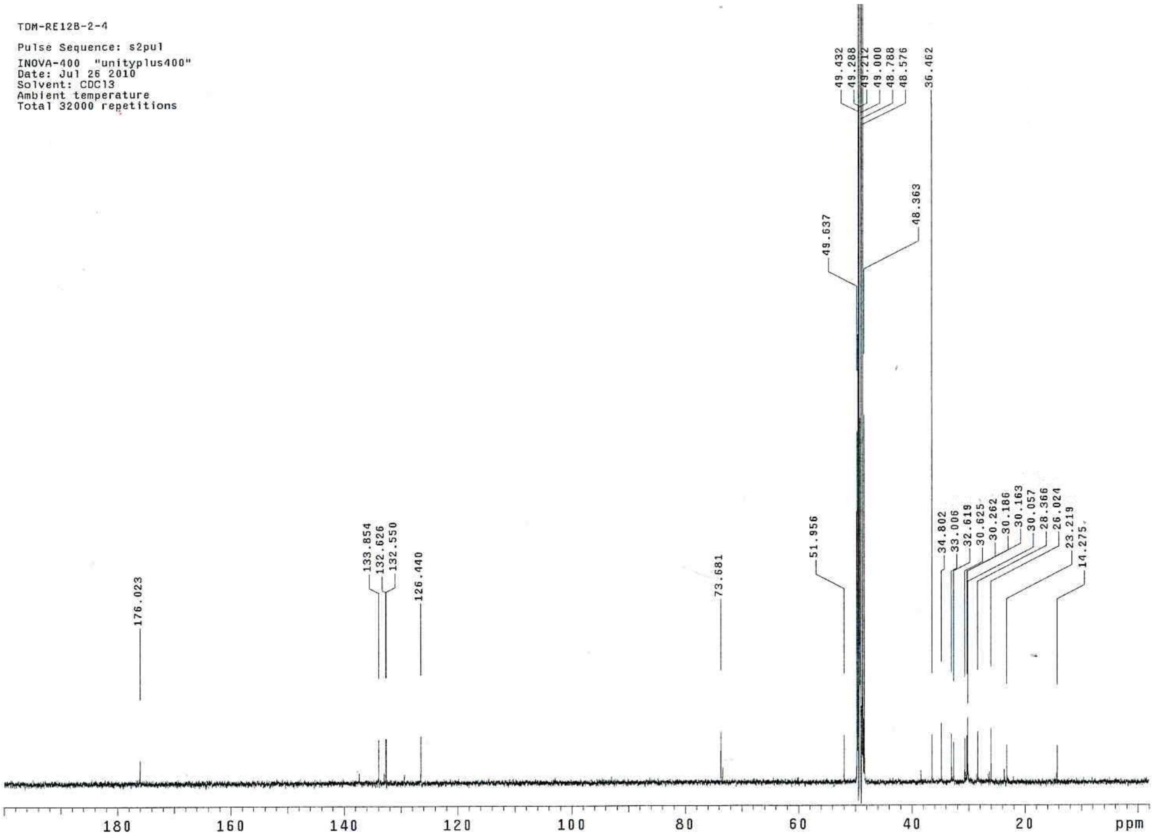

Figure S3. ^{1}H-^{1}H COSY spectrum of compound **1** in CD_3OD

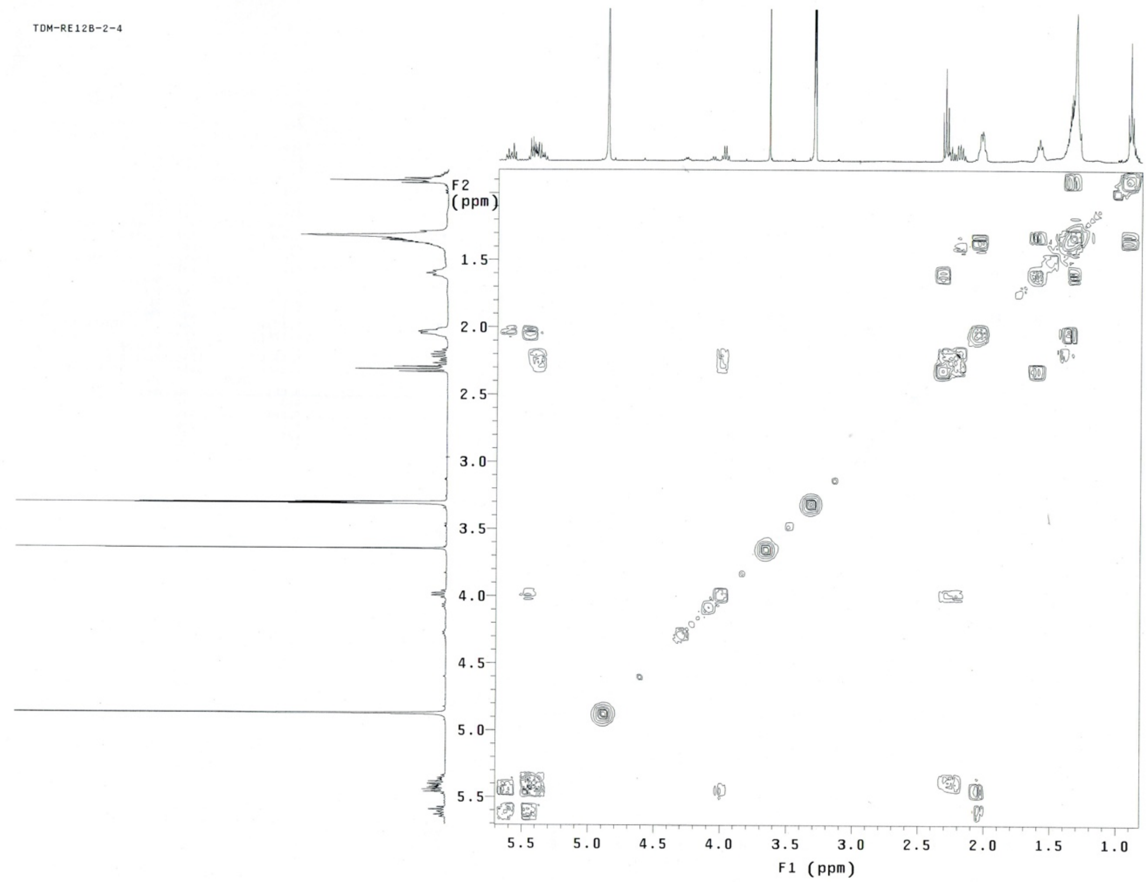

Figure S4. HMQC spectrum of compound **1** in CD_3OD

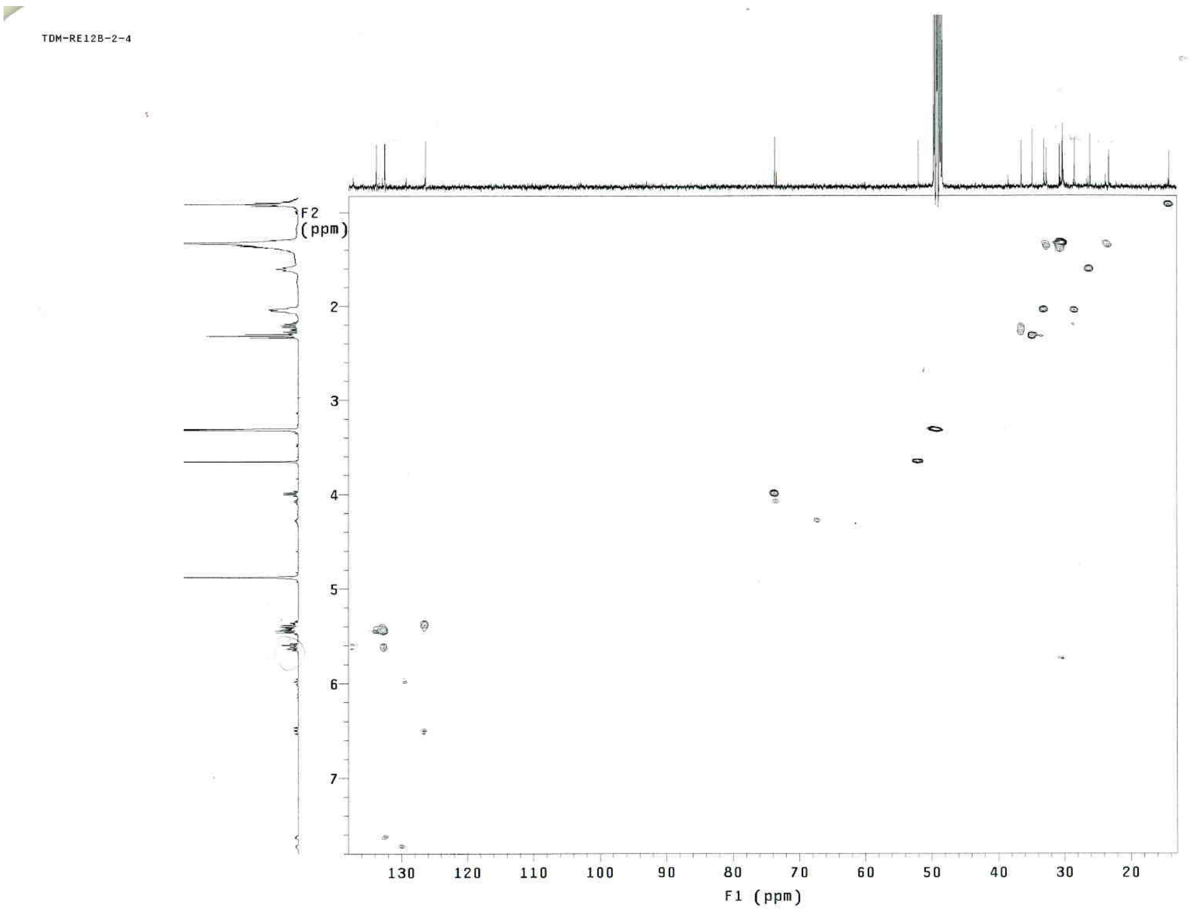

Figure S5. HMBC spectrum of compound **1** in CD_3OD

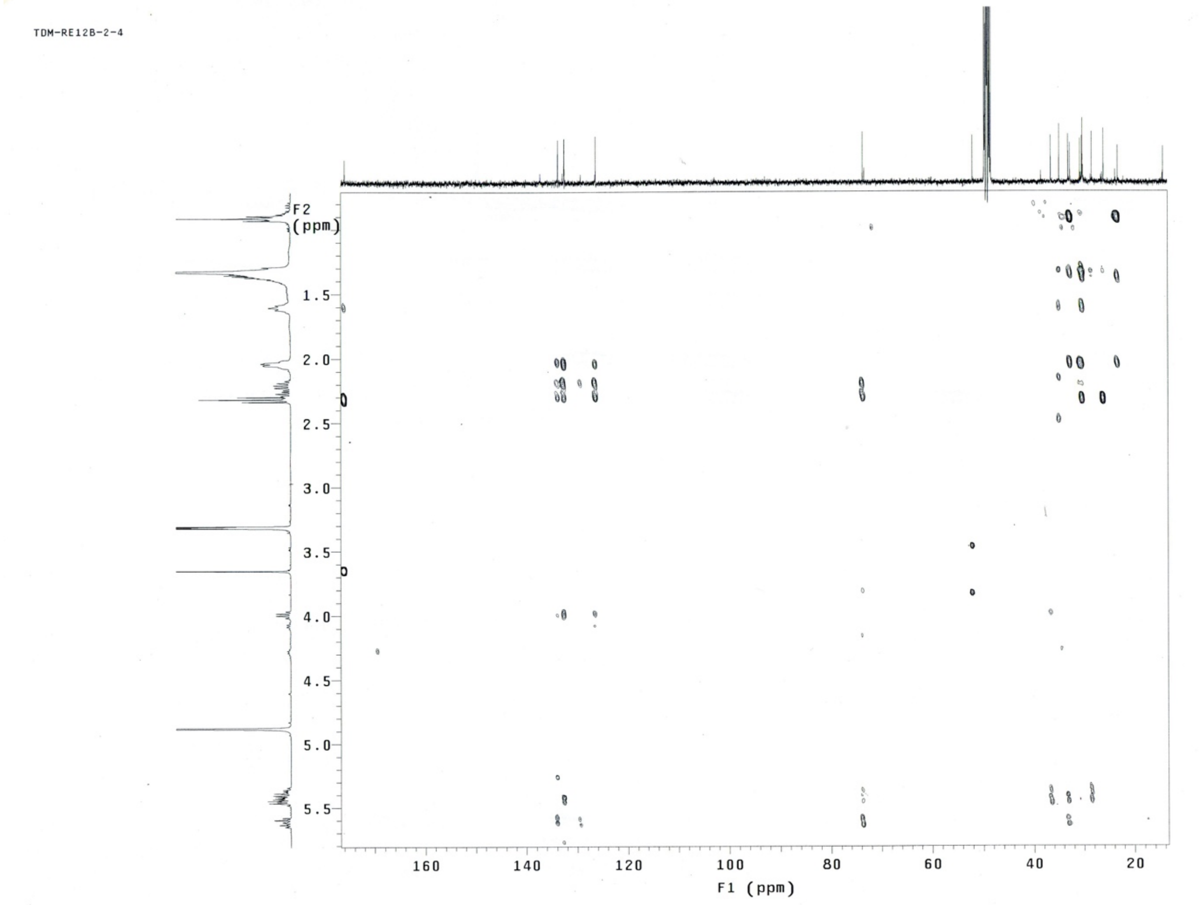

Figure S6. NOESY spectrum of compound **1** in CD_3OD

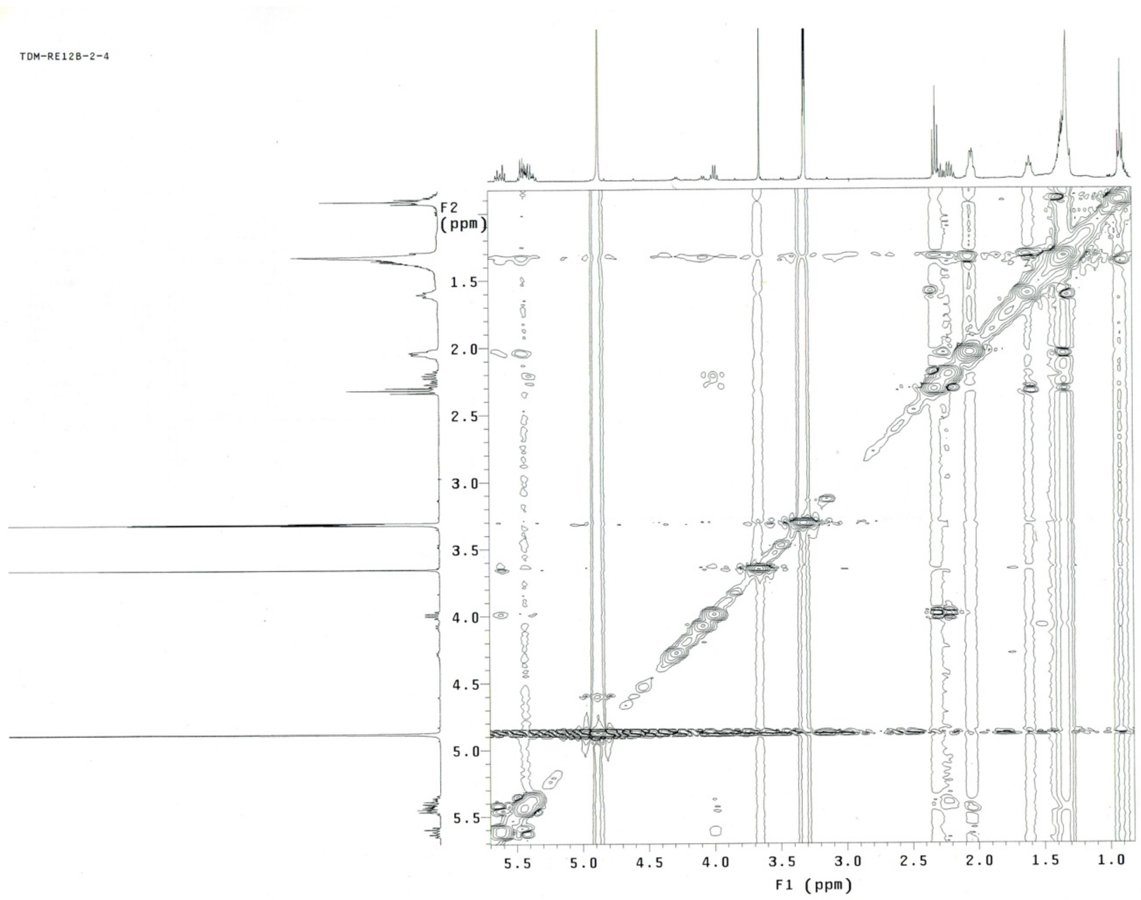

Figure S7. GC-EIMS spectrum of compound **1**

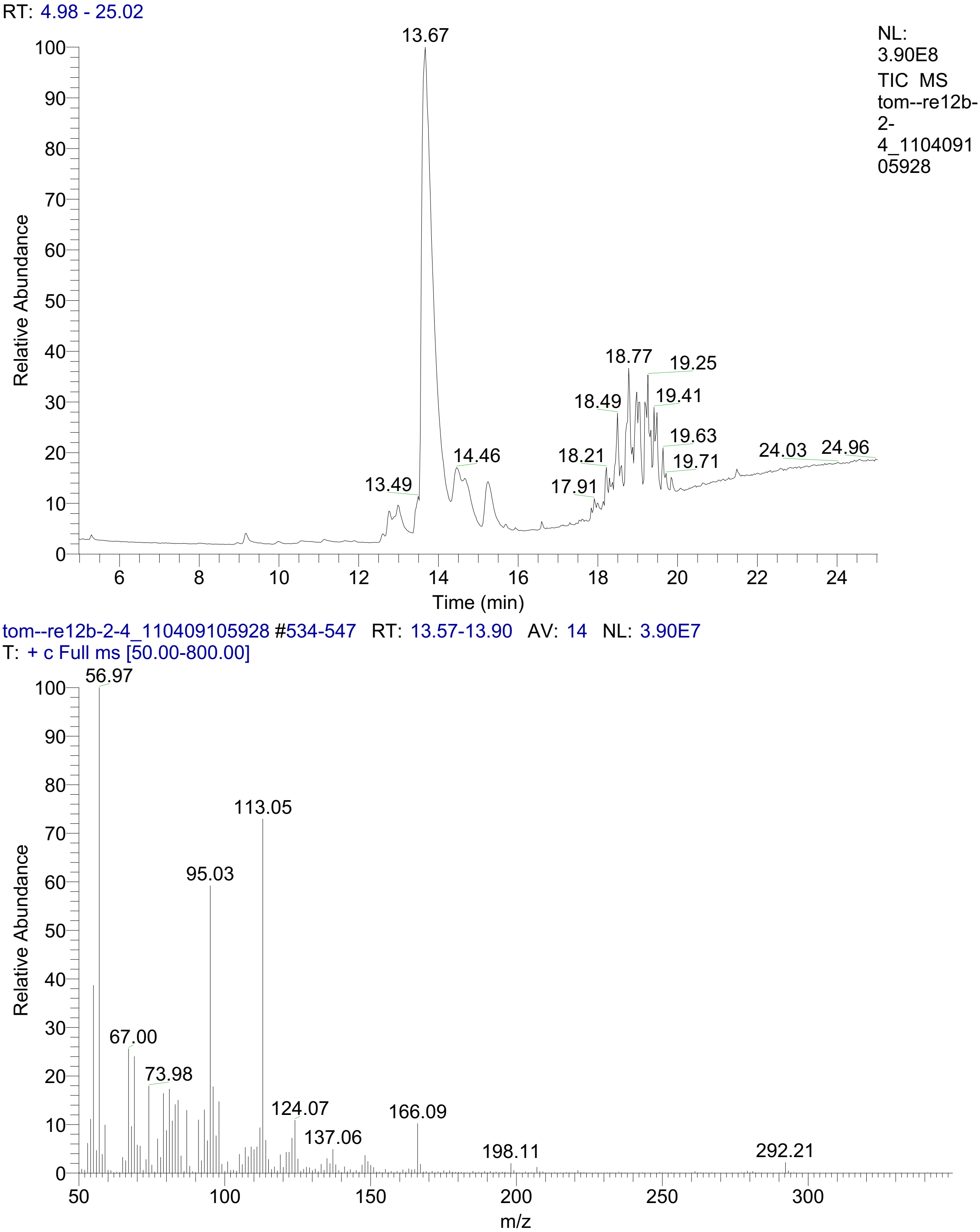

Figure S8. ^{1}H NMR spectrum of compound **2** in CD_3OD, 400 MHz

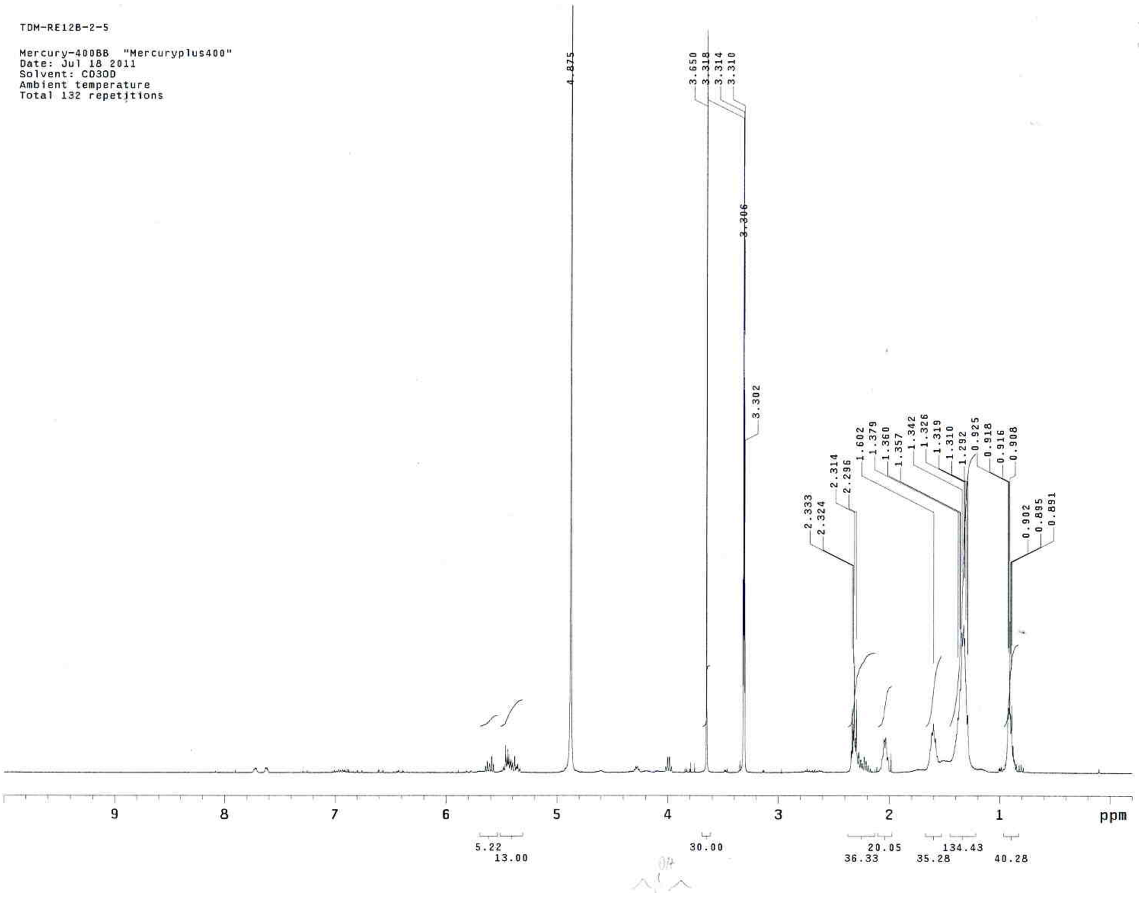

Figure S9. ^{13}C NMR spectrum of compound **2** in CD_3OD, 100 MHz

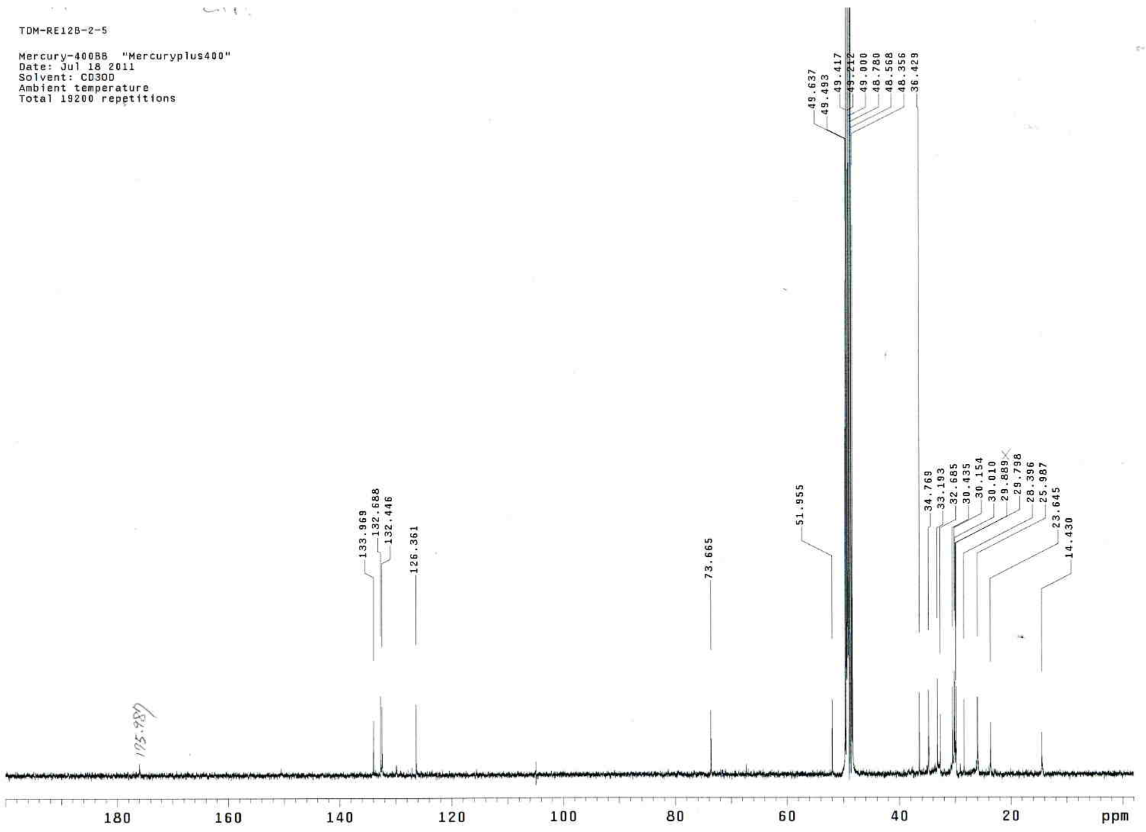

Figure S10. ^{1}H-^{1}H COSY spectrum of compound **2** in CD_3OD

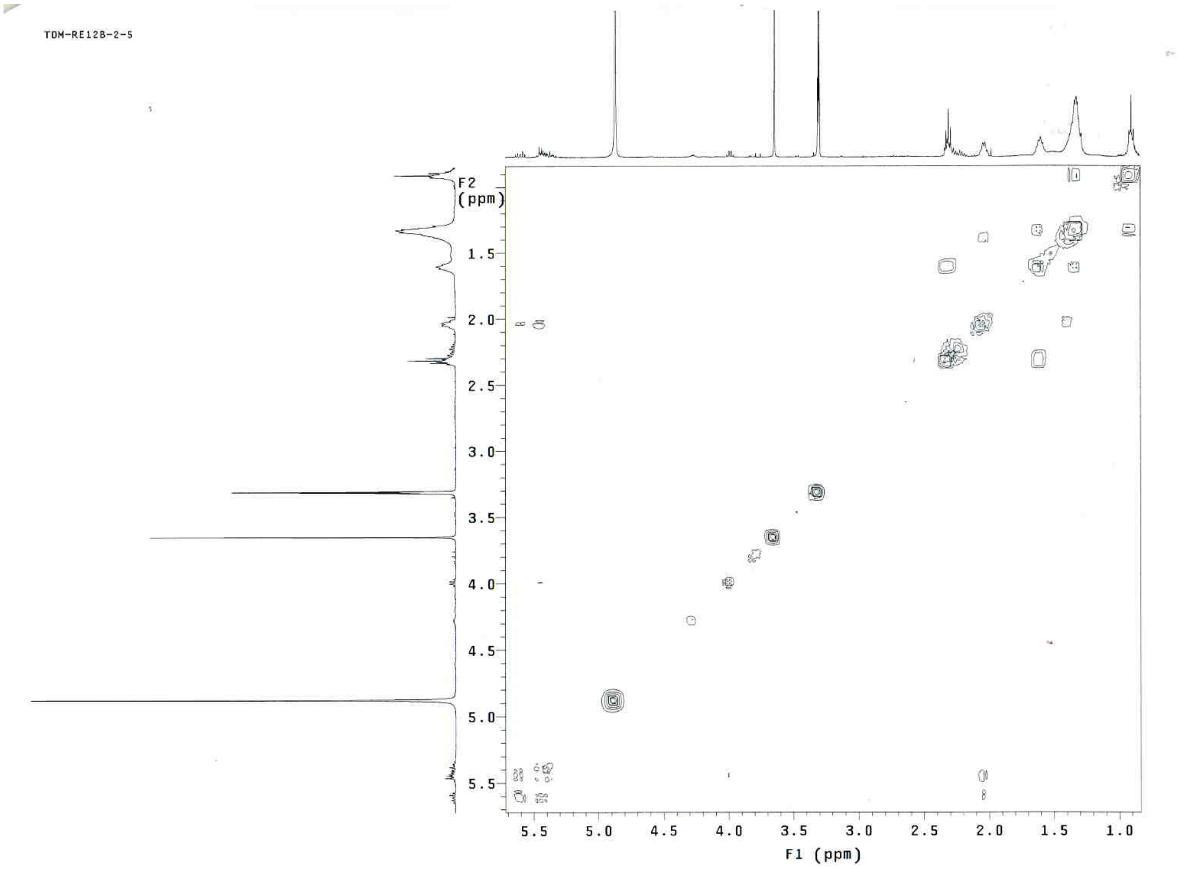

Figure S11. HMQC spectrum of compound **2** in CD_3OD

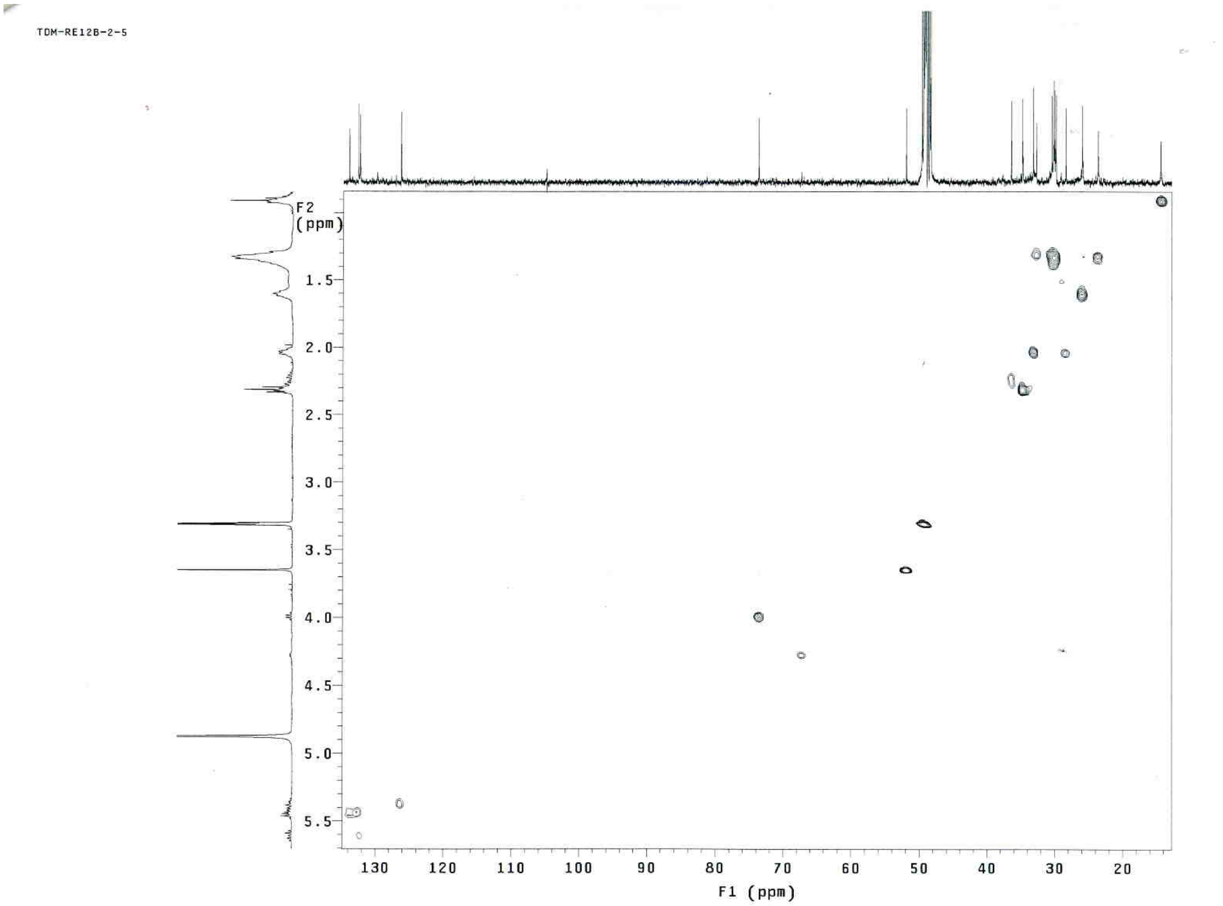

Figure S12. HMBC spectrum of compound **2** in CD_3OD

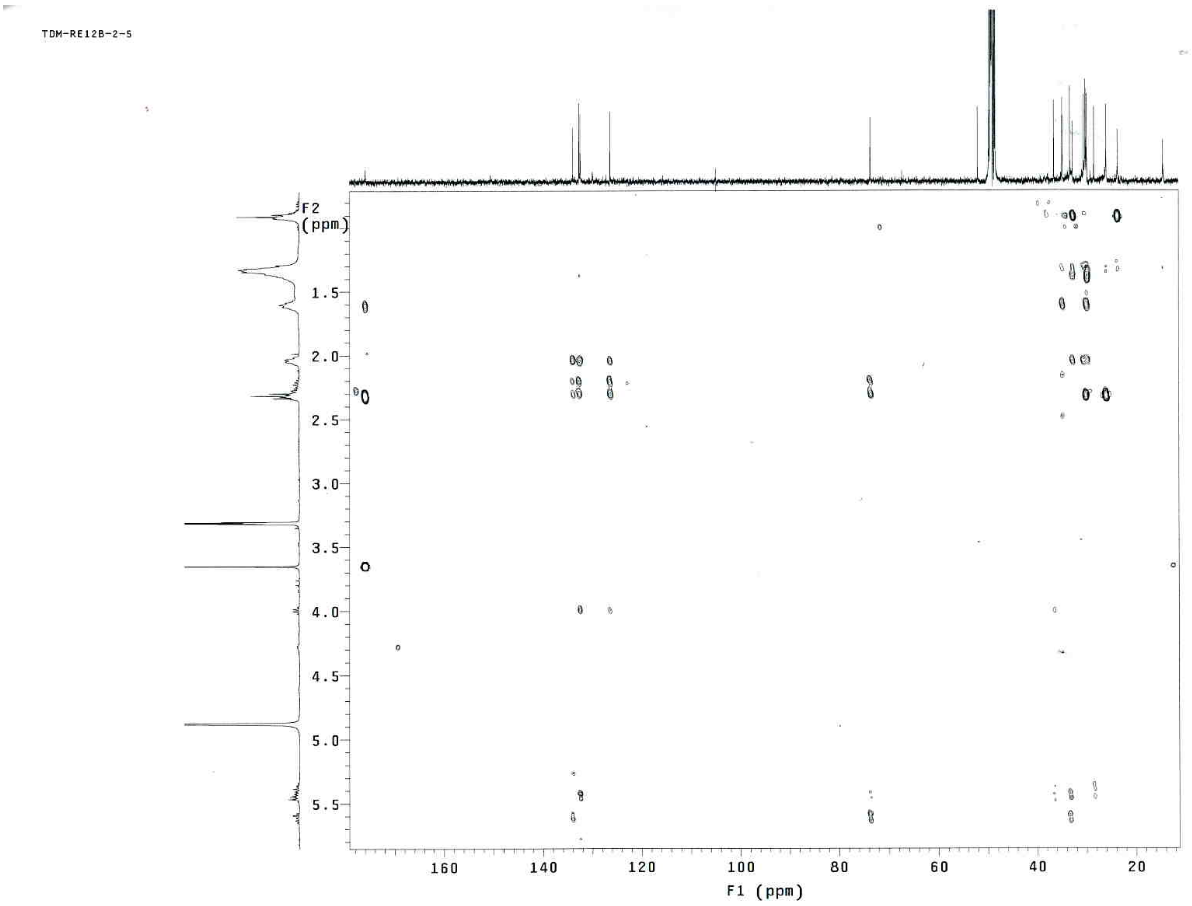

Figure S13. NOESY spectrum of compound **2** in CD_3OD

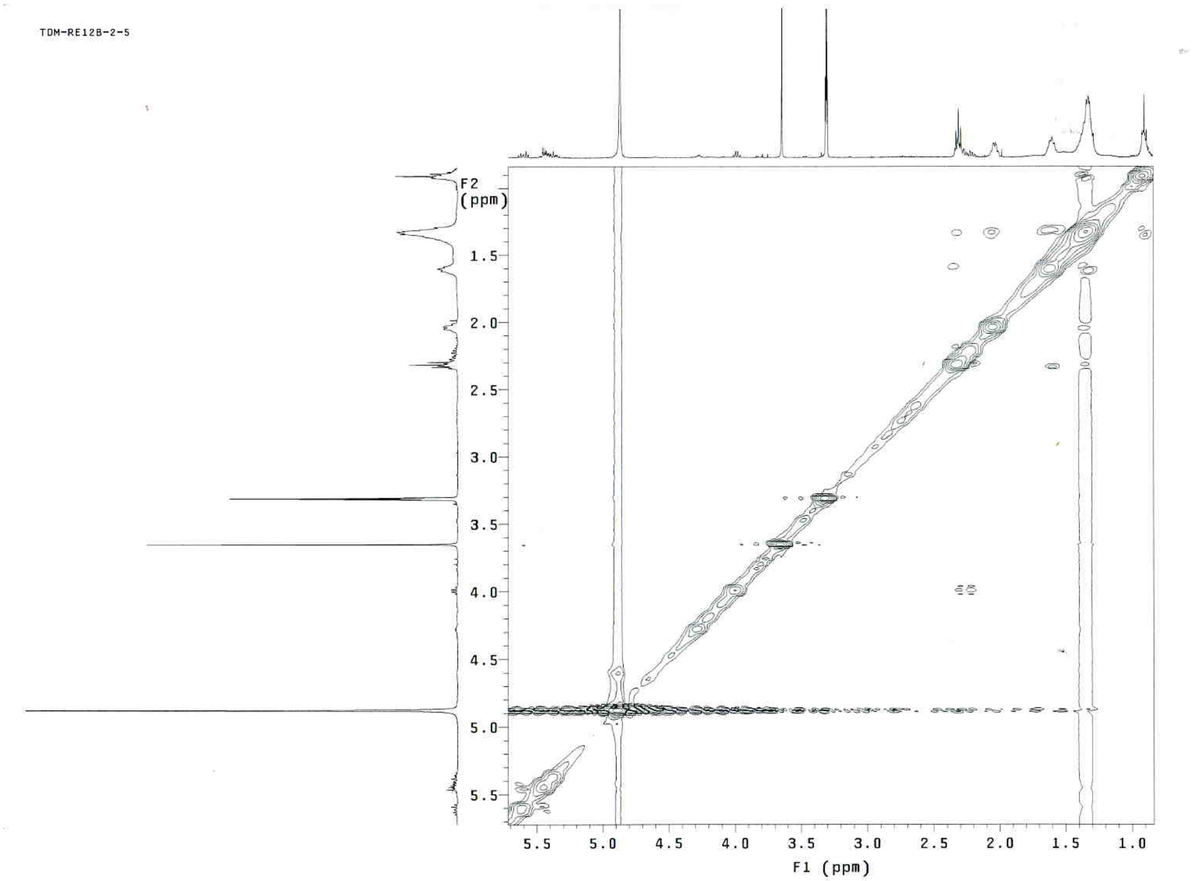

Figure S14. GC-EIMS spectrum of compound **2**

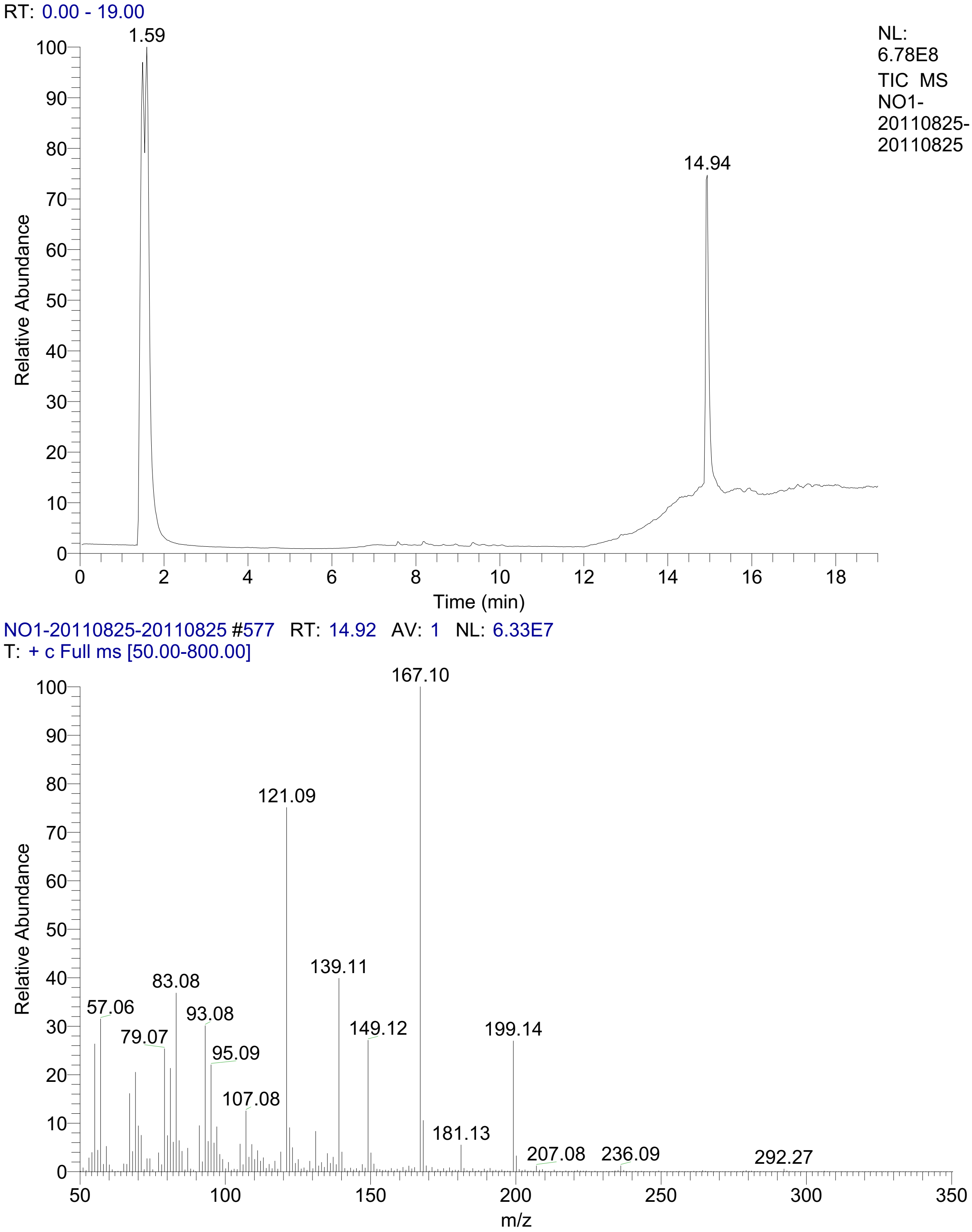

Figure S15. GC-EIMS spectrum of compound **3**

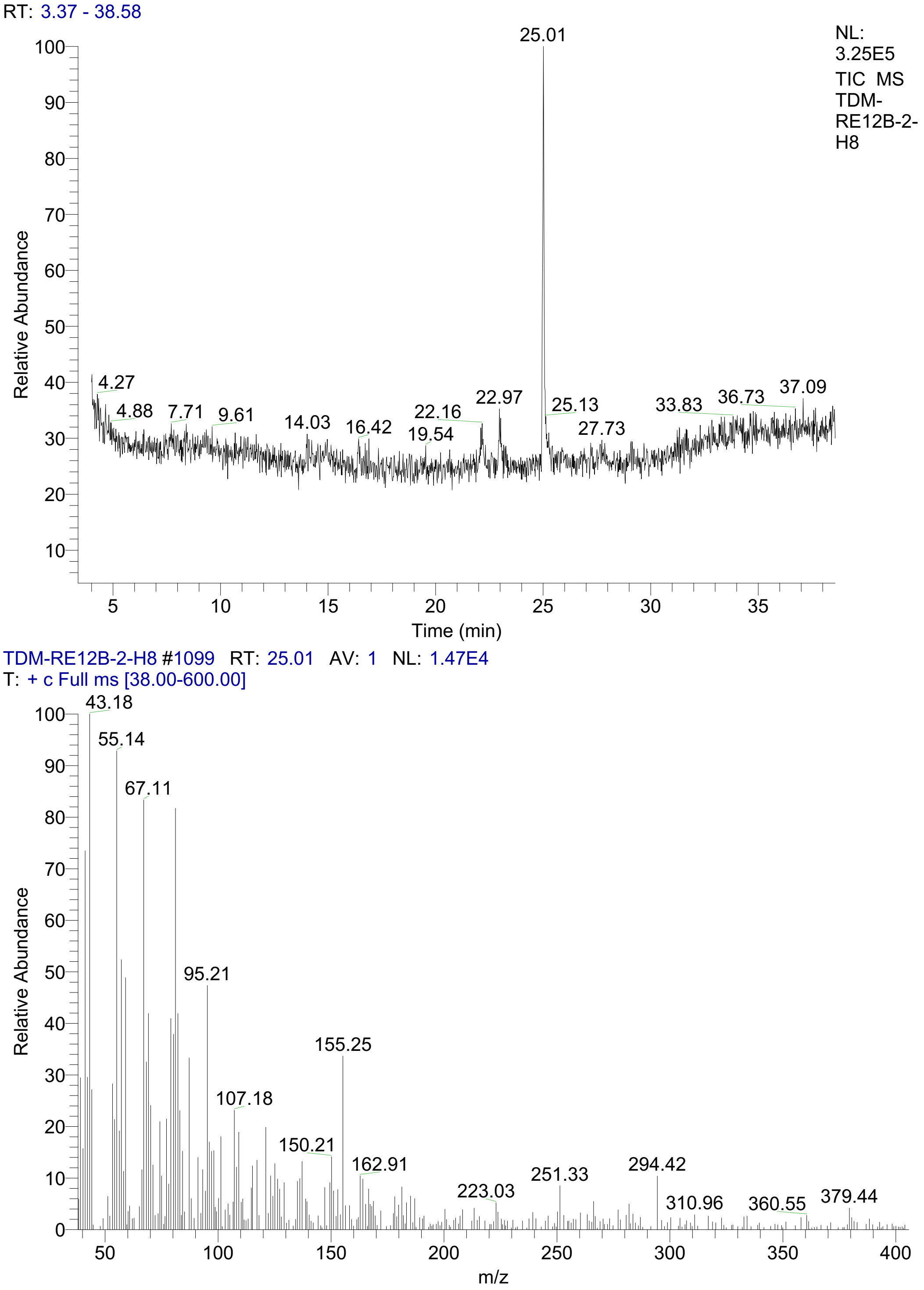

Figure S16. GC-EIMS spectrum of compound **4**

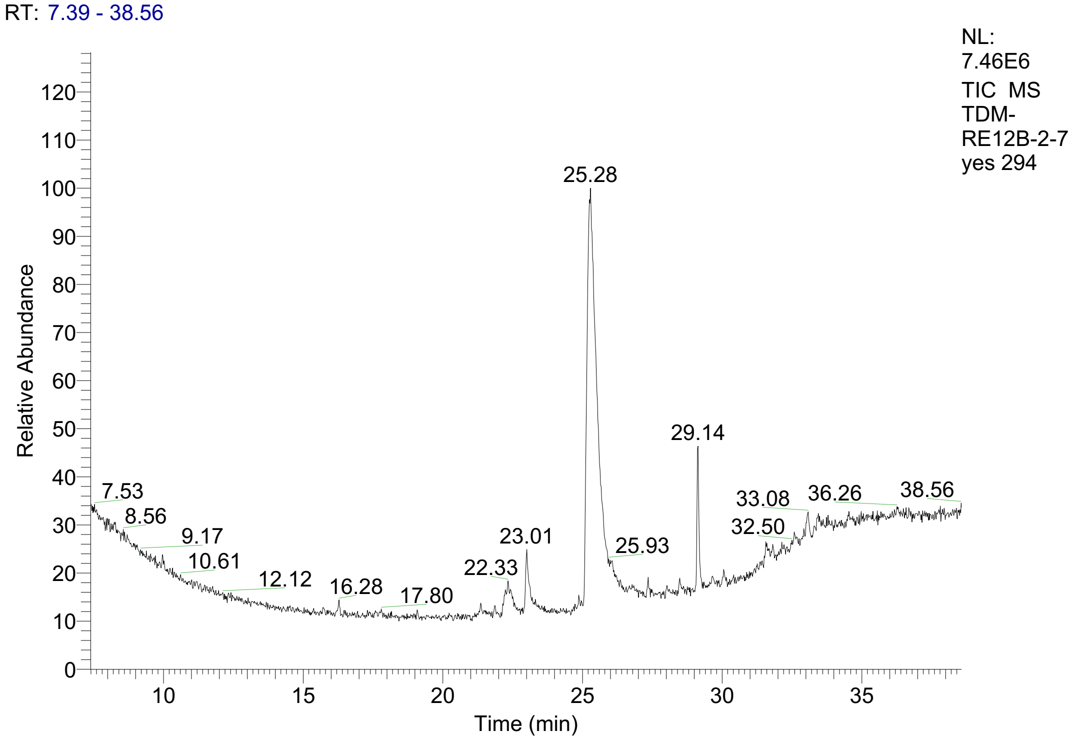

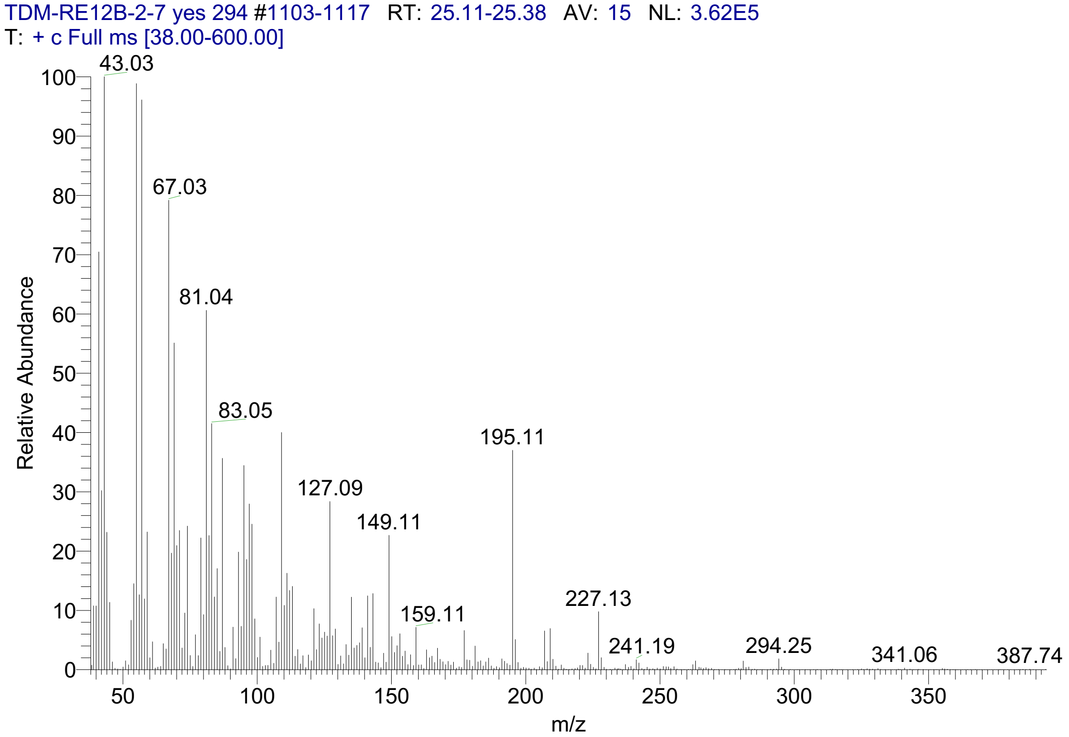

Figure S17. GC-EIMS spectrum of mixture A

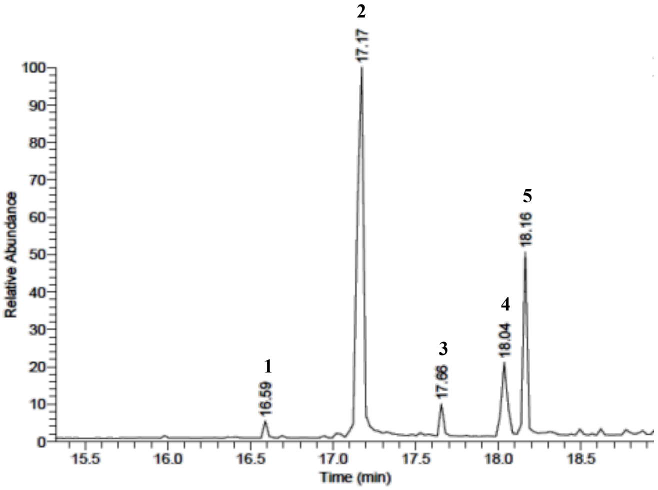

No.	COMPOUND	FORMULA	MW	RT (min)	PERCENTAGE (%)
1	pentadecanoic acid methyl ester	$C_{16}H_{32}O_2$	256.23	16.59	1.91
**2**	**palmitic acid methyl ester**	$\mathbf{C_{17}H_{34}O_2}$	**270.21**	**17.17**	**64.21**
3	margaric acid methyl ester	$C_{18}H_{36}O_2$	284.26	17.67	3.58
4	oleic acid methyl ester	$C_{19}H_{36}O_2$	296.26	18.04	12.25
5	stearic acid methyl ester	$C_{19}H_{38}O_2$	298.25	18.16	18.04

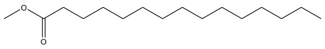

pentadecanoic acid methyl ester

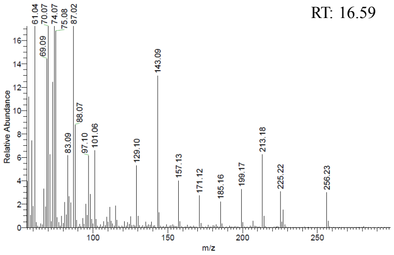

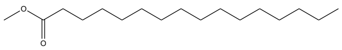

palmitic acid methyl ester

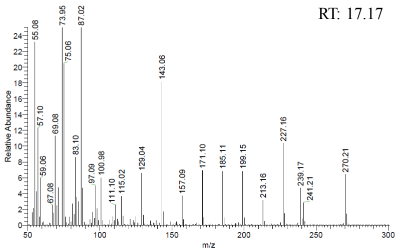

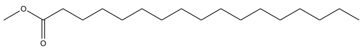

margaric acid methyl ester

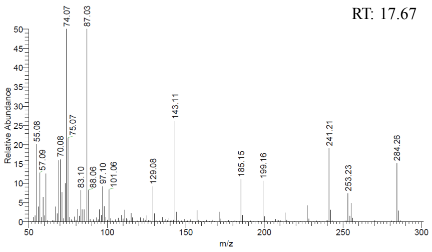

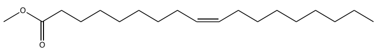

oleic acid methyl ester

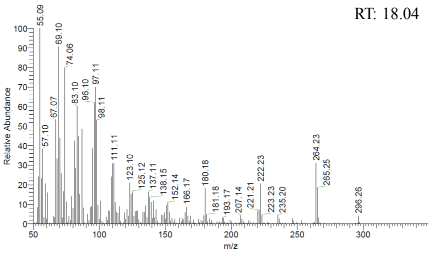

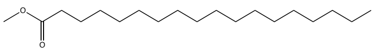

stearic acid methyl ester

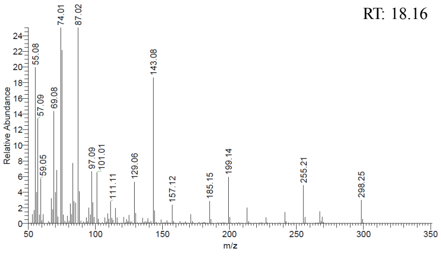

Figure S18. GC-EIMS spectrum of mixture B

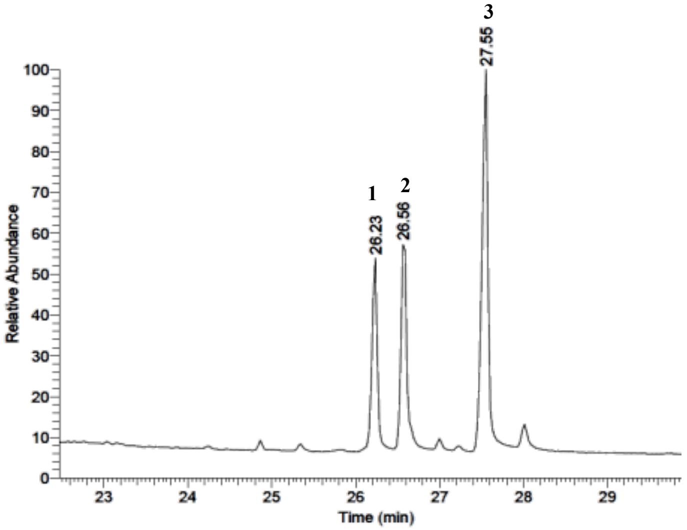

No.	COMPOUND	FORMULA	MW	RT (min)	PERCENTAGE (%)
1	campest-4-en-3-one	$C_{28}H_{46}O$	398.67	26.23	22.75
**2**	stigmast-4,22-dien-3-one	$C_{29}H_{46}O$	410.68	26.56	26.04
3	**β-sitost-4-en-3-one**	$\mathbf{C_{29}H_{48}O}$	**412.70**	**27.55**	**51.21**

1. campest-4-en-3-one

22.75%

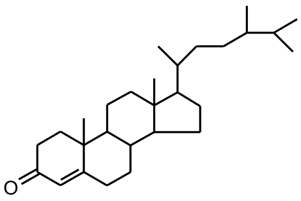

2. stigmast-4,22-dien-3-one

26.04%

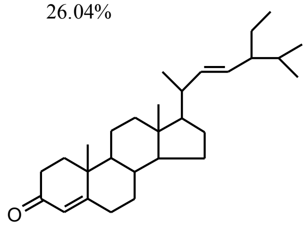

3. β-sitost-4-en-3-one

51.21%

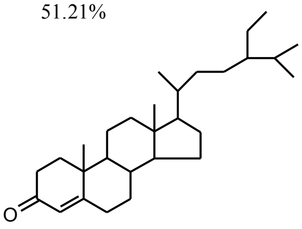

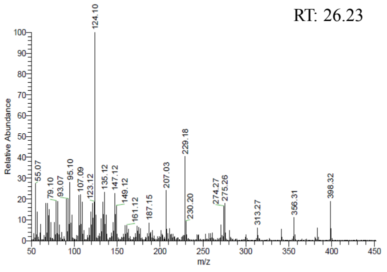

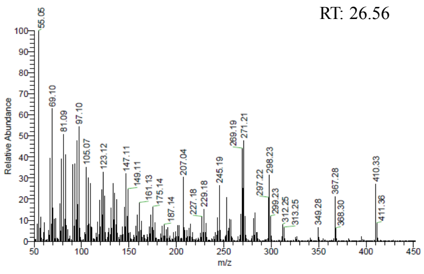

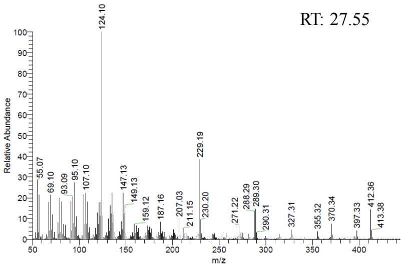

Table S1. Anti-allergic data of fatty acids (1 – 1000 μM). Please see attached xlsx. file.

Table S2. Anti-platelet activity of *Typhonium blumei*

***T. blumei* sample**	**Abbreviation**	**Concentration (μg/ml)**	**Inducer[a]**	
			**Collagen**	**Thrombin**
Leaves crude	TB-L	50	28.2	8.3
Dichloromethane	TB-LD	50	93.6	7.1
Water	TB-LW	50	16.7	22.6
Butanol	TB-LB	50	100.0	100.0
Hexane	TB-LH	50	7.1	4.8
Methanol aq.	TB-LM	50	-14.3	2.4
Rhizomes crude	TB-R	50	17.1	4.8
Ethyl acetate	TB-RE	50	42.9	16.7
Water	TB-RW	50	0.0	-2.4
BuOH	TB-RB	50	65.7	31.0
Hexane	TB-RH	50	22.9	11.9
Methanol aq.	TB-RM	50	14.3	7.1

[a]Platelets were incubated with samples (50 μg/ml) or with vehicle for 3 min, followed by the addition of platelet inducers, collagen (10 μg/ml) or thrombin (0.05 U/ml). Results are expressed as percent inhibition of platelet aggregation (n = 1); compared with the control value (collagen or thrombin). Active fractions are indicated by red color.

Table S3. Cytotoxicity of *T. blumei*

T. blumei sample	Abbreviation	Concentration (μg/ml)	Cancer cell line[a]					
			HepG2[b]	Hep3B[b]	Ca9-22[b]	A549[b]	MCF7[b]	MDA-MB-231[b]
**Leaves crude**	TB-L	20	23.2	27.2	26.1	3.3	21.6	15.8
Dichloromethane	TB-LD	20	49.6	36.2	42.4	25.9	44.3	22.9
Water	TB-LW	20	17.8	19.9	16.4	3.7	4.0	-4.6
Butanol	TB-LB	20	25.7	19.9	18.9	10.2	14.0	5.4
Hexane	TB-LH	20	24.8	21.3	22.3	6.0	17.1	4.3
Methanol aq.	TB-LM	20	49.3	17.9	32.7	12.1	31.8	15.9
**Rhizomes crude**	TB-R	20	5.2	15.1	17.2	5.7	14.5	5.4
Ethyl acetate	TB-RE	20	51.7	17.0	23.7	12.6	31.8	19.1
Water	TB-RW	20	4.1	8.8	5.2	1.2	0.3	0.1
BuOH	TB-RB	20	5.9	8.5	3.6	-0.6	-2.7	-1.8
Hexane	TB-RH	20	3.4	16.9	21.9	-2.1	7.3	2.3
Methanol aq.	TB-RM	20	48.1	16.3	24.4	18.7	29.5	18.0
Doxorubicin		2	93.9	98.0	99.9	86.8	78.5	97.5

[a]Cytotoxicity was evaluated using MTT viability assay; results are presented as percent inhibition of cell growth (n = 1); compared with the control value (DMSO). Active fractions are indicated by red color.

[b]Hep-G2: human hepatocellular carcinoma cells; Hep3B: human hepatocellular carcinoma cells; Ca9-22: oral squamous cell carcinoma cells; A549: human lung adenocarcinoma cells; MCF7: human adenocarcinoma cells; MDA-MB-231: human breast adenocarcinoma cells.

Table S4. Anti-inflammatory activity of *T. blumei*

***T. blumei* sample**	**Abbreviation**	**Concentration (μg/ml)**	**Anti-inflammatory assay**[a]	
			**Superoxide anion generation**	**Elastase release**
**Leaves crude**	TB-L	10	46.7	22.6
Dichloromethane	TB-LD	10	80.0	88.4
Water	TB-LW	10	18.0	10.5
Butanol	TB-LB	10	78.2	84.5
Hexane	TB-LH	10	43.1	74.4
Methanol aq.	TB-LM	10	98.6	95.6
**Rhizomes crude**	TB-R	10	42.1	12.2
Ethyl acetate	TB-RE	10	90.2	93.1
Water	TB-RW	10	3.3	5.4
BuOH	TB-RB	10	97.2	84.6
Hexane	TB-RH	10	89.7	94.1
Methanol aq.	TB-RM	10	99.2	108.7

[a]Anti-inflammatory activity was evaluated by superoxide anion generation and elastase release assays in human neutrophils using fMLF/CB as inducer. Percent inhibition; results are presented as mean ± SEM (n = 3 - 4); compared with the control value (fMLF/CB). Active fractions are indicated by red color.

Table S5. Cytotoxicity of *T. blumei* subfractions

T. blumei **rhizomes ethyl acetate layer**	**Abbreviation**	**Concentration (μg/ml)**	**Cancer cell line**[a]		
			**HepG2**[b]	**A549**[b]	**MDA-MB-231**[b]
TB-RE subfraction 1	TB-1	20	-56.2	4.6	12.7
TB-RE subfraction 2	TB-2	20	-39.6	-2.9	5.6
TB-RE subfraction 3	TB-3	20	-69.4	-0.3	6.6
TB-RE subfraction 4	TB-4	20	-84.1	-1.5	4.1
TB-RE subfraction 5	TB-5	20	-52.4	1.6	3.0
TB-RE subfraction 6	TB-6	20	-88.7	-2.1	-0.9
TB-RE subfraction 7	TB-7	20	33.5	30.9	18.5
TB-RE subfraction 8	TB-8	20	-82.6	-5.6	2.0
TB-RE subfraction 9	TB-9	20	56.8	25.3	23.3
TB-RE subfraction 10	TB-10	20	-27.2	3.9	5.8
TB-RE subfraction 11	TB-11	20	-87.2	-7.7	-4.9
TB-RE subfraction 12	TB-12	20	-21.3	-6.6	-14.8
TB-RE subfraction 13	TB-13	20	-67.5	-16.7	-16.6
Doxorubicin		2	95.2	70.0	73.7
		1	70.1	22.1	31.9
		0.5	58.7	17.3	22.9
		0.25	42.9	16.5	14.7

[a]Cytotoxicity was evaluated using MTT viability assay; results are presented as percent inhibition of cell growth (n = 1); compared with the control value (DMSO). Active fractions are indicated by red color.

[b]Hep-G2: human hepatocellular carcinoma cells; A549: human lung adenocarcinoma cells; MDA-MB-231: human breast adenocarcinoma cells.

Table S6. Anti-inflammatory activity of *T. blumei* subfractions

T. blumei rhizomes ethyl acetate layer	Abbreviation	Anti-inflammatory assay[a]	
		Superoxide anion generation IC_{50} (μg/ml)[b]	Elastase release IC_{50} (μg/ml)[b]
TB-RE subfraction 1	TB-1	>10	3.8 ± 2.2
TB-RE subfraction 2	TB-2	2.0 ± 0.4	2.9 ± 0.5
TB-RE subfraction 3	TB-3	>10	>10
TB-RE subfraction 4	TB-4	>10	>10
TB-RE subfraction 5	TB-5	>10	>10[c]
TB-RE subfraction 6	TB-6	>10[d]	>10
TB-RE subfraction 7	TB-7	>10[e]	6.4 ± 0.9
TB-RE subfraction 8	TB-8	4.3 ± 0.6	2.4 ± 0.5
TB-RE subfraction 9	TB-9	1.5 ± 0.2	1.3 ± 0.1
TB-RE subfraction 10	TB-10	2.5 ± 0.6	1.9 ± 0.6
TB-RE subfraction 11	TB-11	2.6 ± 0.6	1.6 ± 0.3
TB-RE subfraction 12	TB-12	>10[f]	4.2 ± 1.2
TB-RE subfraction 13	TB-13	6.5 ± 1.8	2.3 ± 0.6

[a]Anti-inflammatory capacity was evaluated by superoxide anion generation and elastase release assays in human neutrophils using fMLF/CB as inducer. IC_{50} values; results are presented as mean ± SEM (n = 3 - 4); compared with the control value (fMLF/CB).

[b]IC_{50} values express the concentration of the sample required to inhibit superoxide anion generation or elastase release by 50%.

[c]TB-5 exerted significant inhibitory activity in elastase release (22.9 ±5.6%) assay at 10 μM.

[d]TB-6 exerted significant inhibitory activity in superoxide anion generation (23.2 ± 3.7%) assay at 10 μM.

[e]TB-7 exerted significant inhibitory activity in superoxide anion generation (39.4 ± 1.3%) assay at 10 μM.

[f]TB-12 exerted significant inhibitory activity in superoxide anion generation (14.8 ± 1.4%) assay at 10 μM.

Table S7. Anti-allergic activity of *T. blumei* subfractions

T. blumei rhizomes ethyl acetate layer	Abbreviation	Inhibition of A23187-induced β-hexosaminidase release[a]		
		Concentration (µg/ml)		
		10.0	50.0	100.0
TB-RE subfraction 1	TB-1	—	—	—
TB-RE subfraction 2	TB-2	—	—	30.7
TB-RE subfraction 3	TB-3	—	—	—
TB-RE subfraction 4	TB-4	—	—	27.0
TB-RE subfraction 5	TB-5	—	—	—
TB-RE subfraction 6	TB-6	—	—	—
TB-RE subfraction 7	TB-7	—	—	41.0
TB-RE subfraction 8	TB-8	—	—	—
TB-RE subfraction 9	TB-9	27.7	42.7	TOXIC
TB-RE subfraction 10	TB-10	—	42.0	TOXIC
TB-RE subfraction 11	TB-11	—	—	25.7
TB-RE subfraction 12	TB-12	—	—	—
TB-RE subfraction 13	TB-13	—	—	—
Dexamethasone		80% (50 nM)		

[a] The cytotoxicity of samples to RBL-2H3 was evaluated using MTT viability assay and the toxic concentrations are labelled as 'TOXIC' (viability less than 80%). The inhibition of degranulation was assessed by A23187-induced β-hexosaminidase release in RBL-2H3 cells; percent inhibition; results are presented as mean (n = 3); compared with the control value (A23187). — not active (insignificant inhibition of degranulation, below 20%)
